# Impact of Non-Native Birds on Native Ecosystems: A Global Analysis

**DOI:** 10.1371/journal.pone.0143070

**Published:** 2015-11-17

**Authors:** Valeria L. Martin-Albarracin, Guillermo C. Amico, Daniel Simberloff, Martin A. Nuñez

**Affiliations:** 1 Laboratorio Ecotono, INIBIOMA, CONICET-Universidad Nacional del Comahue, Bariloche, Río Negro, Argentina; 2 Department of Ecology and Evolutionary Biology, University of Tennessee, Knoxville, Tennessee, United States of America; 3 Grupo de Ecología de Invasiones, INIBIOMA, CONICET-Universidad Nacional del Comahue, Bariloche, Río Negro, Argentina; University of A Coruña, SPAIN

## Abstract

Introduction and naturalization of non-native species is one of the most important threats to global biodiversity. Birds have been widely introduced worldwide, but their impacts on populations, communities, and ecosystems have not received as much attention as those of other groups. This work is a global synthesis of the impact of nonnative birds on native ecosystems to determine (1) what groups, impacts, and locations have been best studied; (2) which taxonomic groups and which impacts have greatest effects on ecosystems, (3) how important are bird impacts at the community and ecosystem levels, and (4) what are the known benefits of nonnative birds to natural ecosystems. We conducted an extensive literature search that yielded 148 articles covering 39 species belonging to 18 families -18% of all known naturalized species. Studies were classified according to where they were conducted: Africa, Asia, Australasia, Europe, North America, South America, Islands of the Indian, of the Pacific, and of the Atlantic Ocean. Seven types of impact on native ecosystems were evaluated: competition, disease transmission, chemical, physical, or structural impact on ecosystem, grazing/ herbivory/ browsing, hybridization, predation, and interaction with other non-native species. Hybridization and disease transmission were the most important impacts, affecting the population and community levels. Ecosystem-level impacts, such as structural and chemical impacts were detected. Seven species were found to have positive impacts aside from negative ones. We provide suggestions for future studies focused on mechanisms of impact, regions, and understudied taxonomic groups.

## Introduction

Introduction and naturalization of nonnative species is recognized as one of the most important threats to global biodiversity. Many species have become naturalized worldwide, and many of them affect native ecosystems. Birds in particular have been widely introduced, so that nowadays more than 200 species are naturalized worldwide [1]. Naturalized bird species can negatively affect biodiversity and damage agriculture and human health [2].

Birds are an excellent group for study of invasion patterns and processes. The tight association between birds and people has led to the existence of many records of bird introductions that have allowed tests of several hypotheses about the role of various factors that influence success at each invasion stage: transport, introduction, establishment, and spread [3,4]. The ecological impacts of established non-native birds in novel environments, by contrast, have received less attention than the above-mentioned invasion stages [5]. Nonnative birds may affect ecosystems by different mechanisms and at different levels: population, community, and ecosystem levels. Among described population-level and community-level effects are hybridization with native species, competition, transmission of diseases to native animals, predation of native fauna, and herbivory [5,6]. Among ecosystem-level effects, the introduction of birds may cause eutrophication of water bodies through increased deposition of droppings and changes in plant community composition and vegetation structure through the introduction of novel mutualisms (e.g. pollination and seed dispersal) [6,7].

Ebenhard [8] reviewed the impact of nonnative birds and mammals on natural ecosystems. Of a total of 212 introduced bird species, he found that, in sharp contrast to mammals, only a few had ecological impacts: 3% through competition with native species, 1% through predation, and 5% through other effects. He reported no case of birds changing plant community composition or habitat structure. Shirley & Kark [2], Kumschick & Nentwig [9], and Kumschick et al. [10] more recently reviewed the impact of nonnative birds in Europe on native ecosystems and on the economy. Those studies showed that the degree of bird impact differs between taxonomic groups and is associated with specific traits. Kumschick & Nentwig [9] found that species of the families Anatidae and Psittacidae had the highest impact on biodiversity. Also, highest impacts on biodiversity and the economy can be associated with species of large body mass, habitat breadth, and geographic range [10]. A comparison of traits associated with impact between Europe and Australia found that the only specific trait consistently associated with all measures of impact is generality of habitat use [11]. Impacts revisited among previously mentioned studies on introduced birds include those on native ecosystems—competition, hybridization, predation, herbivory, transmission of diseases to wildlife, and eutrophication of water bodies—and economic impacts.

The impact of introduced birds on native species and biodiversity has not been studied as thoroughly as the impact of other taxonomic groups, and studies usually focus on impacts at the population level [5]. Little is known about the importance of nonnative bird impacts at the community and ecosystem levels [9], and there is currently debate about the importance of nonnative bird impacts and the need to conduct eradication campaigns [9,12]. It is therefore relevant to study in depth the global impact of nonnative birds on native ecosystems.

This analysis surveys studies of the impact of nonnative birds on native ecosystems to see what is known and what direction the next studies should take. Specifically, we want to know (1) what regions of the world, which families, and which mechanisms of ecological impact are most represented in the literature, (2) which families and species have the greatest ecological impact, (3) which are the most severe impacts at the population and community levels, (4) whether birds can significantly affect entire ecosystems, causing chemical, physical, or structural changes, and (5) how many species are reported to confer benefits and how relevant they are in comparison to their negative impacts.

## Methods

The list of naturalized nonnative bird species of the world—here, naturalized species are considered species that have been imported to a new country either deliberately or accidentally by human agency, and that are currently established in the wild in self-perpetuating populations independently of humans—was taken from Lever [1] and completed with species present in Long [13], in the ISSG Database (http://www.issg.org/database/welcome/), and with a general literature search, as explained below. This procedure yielded a final list of 213 species in 46 families (S1 File).

We conducted an extensive literature search of studies that addressed the impact of naturalized bird species on native ecosystems. Sources searched were the Scopus database and Google Scholar. Terms used in the search were “(species name)” + “non-native”, “naturalized” and “invasive” + “impact” and “effect” + “competition”, “hybridization”, “predation”, “disease transmission”, “seed dispersal”, “herbivory”, and “eutrophication”. Some extra references were taken from Lever (2005) and Long (1981) [1,13]. We selected those papers that presented conclusions based on research data or that reported observational data. The list of analyzed species included not only birds introduced and naturalized in novel areas but also hybrid birds bred in captivity and released as part of restocking programs [14,15].

The impact of species on native ecosystems was evaluated by using a descriptive scoring system (Adapted from [9]; see S1 File). Only statistically significant results from the literature were taken as evidence of population, community, or ecosystem-level effects. Anecdotal observations were taken into account but were considered as affecting only individual fitness. This approach accords with the precautionary principle, because when there is some evidence of negative impact for an introduced species, although minimal, the species is considered to have an impact. Using the information collected, we assigned each naturalized species a score from 0 (no impact detected) to 5 (massive impact) for every category of impact. A zero was assigned when the information available reported no impact; if we did not find information, we categorized it as “no data”. In cases in which the score was between two values (e.g., between 2 and 3), the average score (e.g., 2.5) was assigned. This is a modified version of the Kumschick and Nentwig´s scale [9].

Kumschick and Nentwig´s scale was chosen because it allows a wide differentiation of the degree of impact (six levels for each impact category) and because it is easily applied when information comes from several different sources. This scoring system has been applied previously to evaluate impacts of nonnative birds in Europe and Australia [9,11]. For this study, Kumschick and Nentwig´s scale was extended following the unifying classification recently proposed by Blackburn et al. [16], and to the six existing categories (Herbivory, Competition, Predation, Transmission of diseases to wildlife, Hybridization, Impact on ecosystem) a novel category was added, “Interaction with other non-native species”. Impact scores for hybridization were slightly modified to achieve a better differentiation of impacts between species, and following Blackburn et al. [16], the category “Herbivory” was renamed “Grazing/Herbivory/Browsing” and the category “Impact on ecosystems” was renamed “Chemical, physical, or structural impact on ecosystem” (see S1 File). Other categories of impact listed in Blackburn et al. [16] were not detected for introduced birds.

Studies were classified according to where they were conducted. Following Lever [1], we considered nine regions: Africa, Asia, Australasia, Europe, North America, South America, Islands of the Indian Ocean, Islands of the Pacific Ocean, and Islands of the Atlantic Ocean. Many species had been introduced in more than one region. The number of separate introductions was 469. An impact score was assigned for each region where the species’ impact was studied. The impact of a species at any one region was assigned as the maximum value of impact found across all categories [16]. Global impact of the species was calculated as the sum of its impact scores across all regions. Average impact of each family is the mean of global species impact for all species in that family, and global impact of the family is the sum of global species impacts for all species in the family. Possible benefits of species for biodiversity were recorded but were not included in the scoring system. To compare the relative importance of each impact category, the average score of each one was calculated across all species and regions.

Because the greater a species’ residence time, the greater the area in which it is established, impacts detected can be biased by the introduction date [17]. To evaluate this possible bias, we calculated the correlation between the impact of every species and its introduction date. In addition, we calculated the mean residence time and its standard error for species with ecological impacts for every category having at least 7 data points (competition, interactions with other alien species, hybridization and transmission of diseases).

To study the validity of using this scoring system as a tool to study impacts, we evaluated the repeatability of this procedure by comparing our results with those obtained by Strubbe *et al*. [12] for shared species in Europe between both works. To this end we applied Wilcoxon signed-rank tests for paired comparisons.

## Results

A total of 148 articles were obtained for 39 species belonging to 19 families, representing 18% and 41% of species and families naturalized, respectively. The list of naturalized species and families and their statuses (studied or unstudied) is provided in S1 Table. The complete list of articles analyzed is provided in S2 File. Eleven species have been studied in more than one region. Scores assigned to species for every impact category are presented in S2 Table.

**Fig 1:**
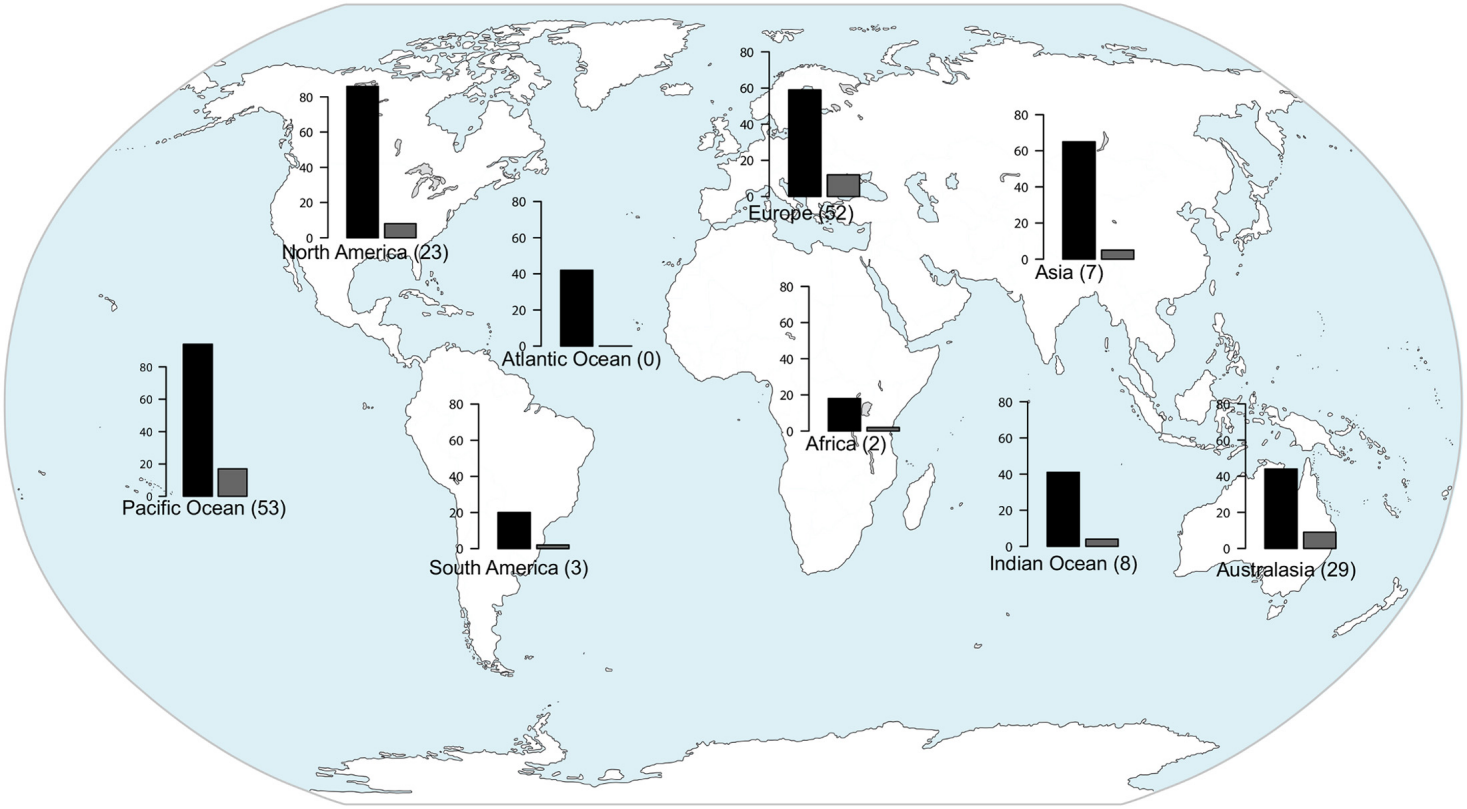
Naturalized and studied number of species in each region. Number of naturalized species (black bars) and of studied species (grey bars) in the nine regions considered. Numbers between parentheses indicate the number of articles analyzed.

The majority of studies were conducted in Europe (33%) and on islands of the Pacific Ocean (21%). Regions where the impact of nonnative birds has been less studied are islands of the Atlantic Ocean, Africa, and South America (Fig 1). The proportion of studied species in relation to the total number of introduced species per region was greater in Australasia, followed by Europe and islands of the Pacific Ocean (Pearson´s Chi-squared test, p = 0.03).

Families most studied were Sturnidae (21% of studies, 4 species out of 10 introduced) and Phasianidae (20%, 8 species out of 25). Families with the highest proportion of studied species were Turdidae and Zosteropidae (only families with more than one species introduced were considered; Pearson´s Chi-squared test, p<0.01).

**Fig 2:**
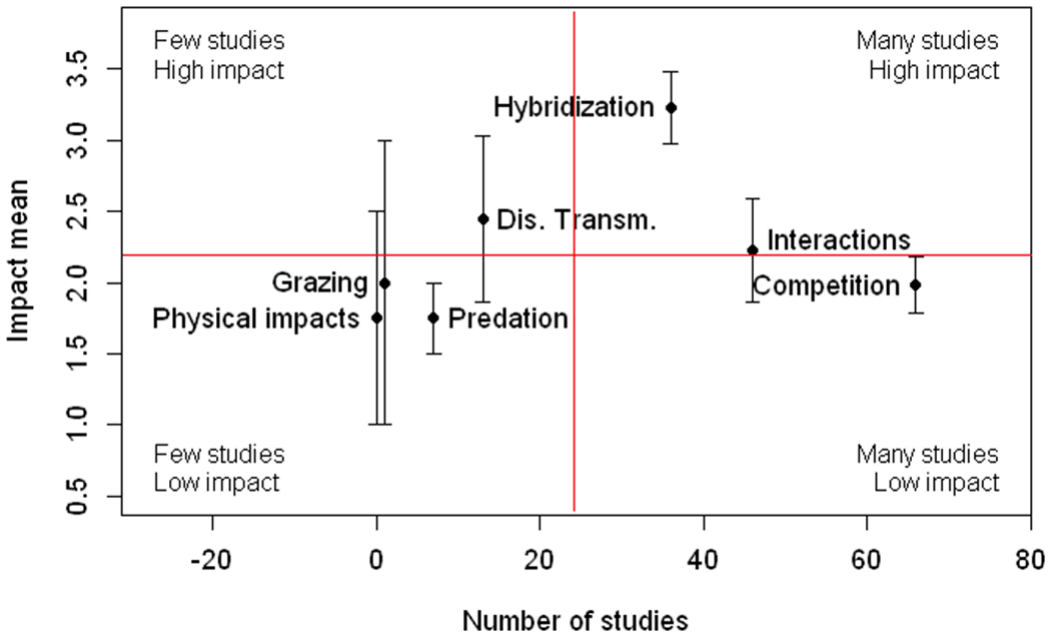
Relation between mean impact value and the number of studies conducted for every impact category. Number of studies conducted for every impact category (x axis) and the corresponding impact mean (y axis). Bars represent the standard error. Vertical and horizontal lines are located at the mean of x and y axes respectively and separate points in four quadrants according to the relative number of studies and the level of impact. Categories “chemical, physical or structural impact on the ecosystem”, “grazing/herbivory/browsing”, and “interaction with other non-native species”, are abbreviated to “physical impacts”, “grazing”, and “interactions”, respectively.

**Fig 3:**
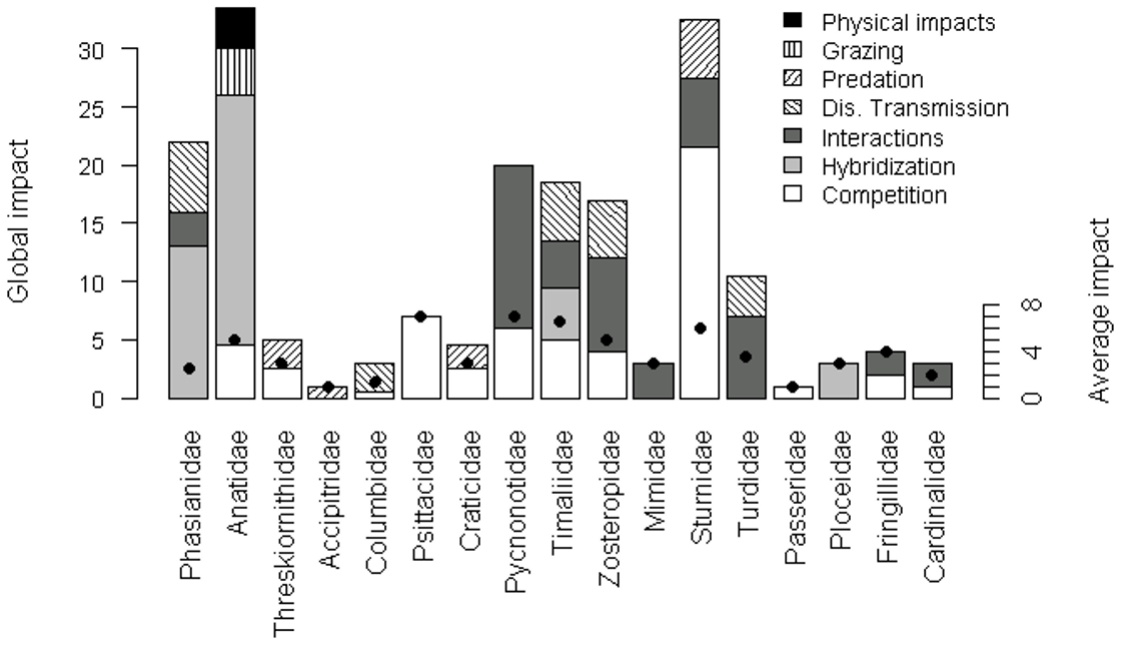
Impacts of avian families on natural ecosystems. Bars represent global impacts of the family and black points the average impact of the family. Only families with reported impacts are shown. Categories “chemical, physical or structural impact on the ecosystem”, “grazing/herbivory/browsing”, and “interaction with other non-native species”, are abbreviated to “physical impacts”, “grazing”, and “interactions”, respectively.

The impacts most studied were competition (39% of studies), interaction with other non-native species (27%), and hybridization (21%) (Fig 2). The impacts least studied were grazing/ herbivory/ browsing and chemical, physical, or structural impact on ecosystem. A test for differences of scores by category of impact was nearly significant (Kruskal-Wallis rank sum test, p = 0.07). Hybridization with native species was the category with a highest score (average 3.23), followed by disease transmission (average 2.44) and interaction with other non-native species (average 2.23). The other four categories had scores equal to or less than 2.00 (Fig 2). Competition and interaction with other non-native species were the most taxonomically widespread impacts (detected for 12 and 9 families respectively, Fig 3). Hybridization was detected in 4 families, disease transmission in 5, and predation in 4 families. Chemical impact on ecosystem and grazing/ herbivory/ browsing were detected only for Anatidae (Fig 3). We found reports of negative impacts for 35 species and of positive impacts for only 7 species. For those seven species, negative and positive impacts are described in Table 1. Only one species had only positive impacts reported.

**Table 1:** Negative and positive impacts of the few bird species with reported benefits in the bibliography.

Species	Negative impact	Positive impact
Ring-necked Parakeet (*Psittacula krameri*)	Impact = 2. Competition with native birds for nesting cavities.	May increase the number of available cavities in urban parks of Germany, owing to their ability to enlarge smaller cavities and excavate new cavities in soft-wooded trees (Czajka, et al. 2011).
Japanese White-eye (*Zosterops japonicus*)	Impact = 4 (competition), 3.5 (interaction with other non-native species), 5 (disease transmission). Exploitative competition with native, endemic birds causing population declines. Seed dispersal of both native and non-native plants, predation of seeds of one native species. Reservoir for the malarian parasite.	Interspecific learning: in sympatry with this species, the native Ogasawara Islands Honeyeater is more adept at eating novel foods (Kawakami and Higuchi 2003). One of the main seed dispersers in Hawaii, where the majority of native dispersers have become extinct and remaining ones are scarce (Foster and Robinson 2007). Key seed disperser in Bonin islands, where the set of native dispersers is impoverished. (Kawakami, Mizusawa and Higuchi 2009).
Australian Magpie (*Gymnorhina tibicen*)	Impact = 2.5 (competition), 2 (predation). Aggressive bird that competes with native birds by attacking them. Nest predator of native birds.	May promote the abundance of some species by harassing the Harrier, a major avian predator (Morgan, et al. 2005).
Red-billed Leiothrix (*Leiothrix lutea*)	Impact = 1 (competition, Asia), 2 (competition, Is. Pacific Ocean), 2 (interaction with other non-native species), 5 (disease transmission). Apparent competition with native birds through shared predators. Seed dispersal of both native and non-native plant species. Reservoir for the malarian parasite.	One of the main seed dispersers in Hawaii, where the majority of native dispersers have become extinct and remaining ones are scarce (Foster and Robinson 2007).
Common Pheasant (*Phasianus colchicus*)	Impact = 2 (interaction with other non-native species), 2 (disease transmission). Seed dispersal of both native and non-native plant species. Apparent competition with native species through shared parasites.	Seed dispersal of native plants whose dispersers are extinct or very scarce (Cole, et al. 1995).
Sacred Ibis (*Threskiornis aethiopicus*)	Impact = 2.5 (competition), 2.5 (predation).	Preys on invasive Red Swamp Crayfish (Marion 2013)
Golden Pheasant (*Chrysolophus pictus*)	Impact = 0 (competition).	The species has no positive impacts reported, but the naturalized population in Britain may be important for conservation of the species at a global level, because it is declining in its native habitat (Balmer, et al. 1996).

Table showing the negative and positive impacts for all species with reported benefits in the bibliography. The scores obtained for negative impacts based on the impact scale are given and positive impacts are described.

Our analysis did not reveal significant differences in species scores of global impact between families (Kruskal-Wallis rank sum test, p = 0.17). Species with highest global impact were *Anas platyrhinchos* (16), *Acridotheres tristis* (13) and *Pycnonotus jocosus* (10), and families with highest global impact were Anatidae (score 25), Sturnidae (24) and Phasianidae (20) (Fig 3).

**Fig 4:**
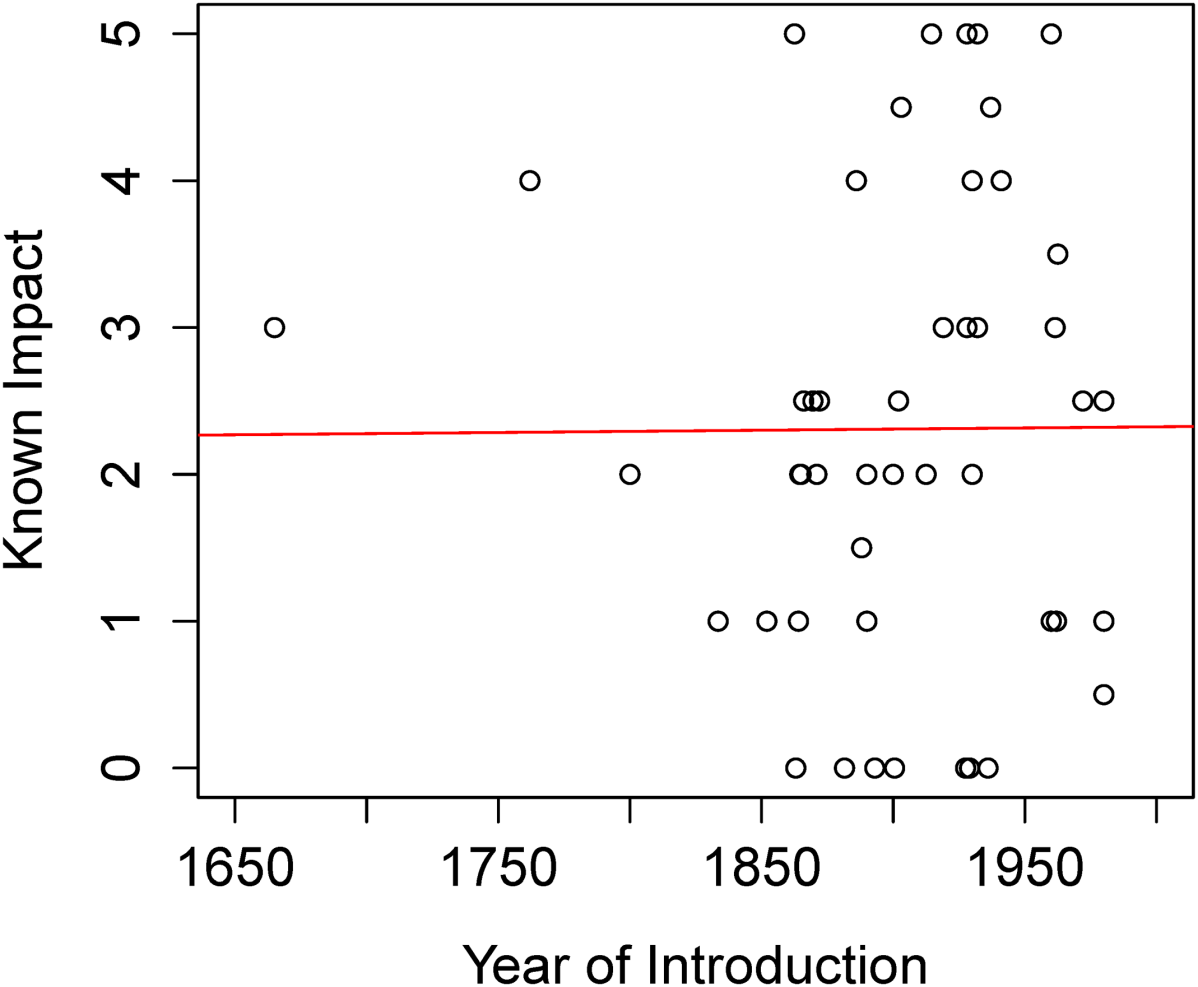
Relation between known impact of a species and its residence time. Each point represents the event of introduction of one species to one region of the world. Multiple R-squared: 3.30e-05, Adjusted R-squared: -0.02, p = 0.97.

**Fig 5:**
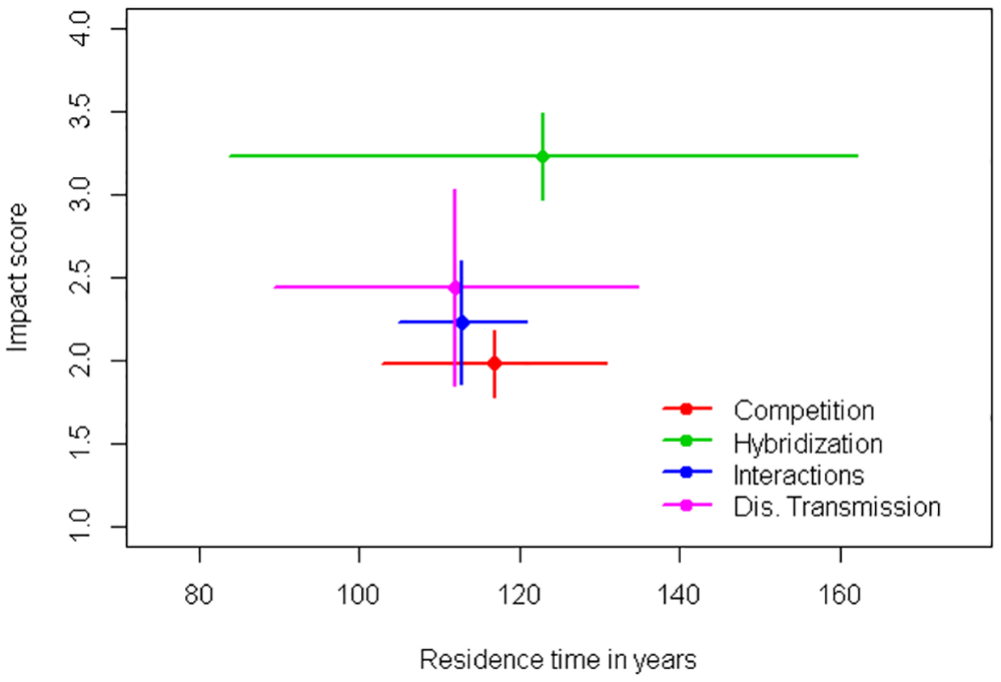
Relation between mean impact score by category and mean residence time. Points represent the mean values and error bars the standard error. This graph shows only the four categories that have at least seven data points.

Linear regression showed no correlation between year of species introduction and impact scores (adjusted R-squared: -0.02, p = 0.97; Fig 4). Mean residence time was similar among impact categories (Kruskal-Wallis Test, p = 0.87). Results are shown in Fig 5. We found no differences between impact scores of Strubbe *et al*. and ours (Wilcoxon test, p = 1).

## Discussion

Analyses showed that geographical differences in number of species studied exist, with Australasia, Europe, and Islands of the Pacific Ocean the regions with highest proportion of studied species in relation with their number of naturalized species. The relative abundance of data on impact from those regions has allowed scientists to do the first syntheses of introduced bird impacts [10,11]; but to achieve global generalizations, it will be necessary to promote research in less studied regions (i.e., islands of the Atlantic Ocean, Africa, and South America). Research on these continents would also allow us to compare impacts of nonnative species between regions with very different histories of human colonization and degrees of urbanization. Much of the available information on impacts of nonnative birds comes from Europe, a continent highly modified by humans for a very long time [18], but impacts on biodiversity could be more pronounced in regions with a greater proportion of native, pristine habitats.

The attention given to each category of impact (measured as number of published studies) was not correlated with its average score (Fig 2), showing that some relatively highly-scored impacts that have been understudied should be prioritized for research. Transmission of diseases to native fauna, for example, can have high impacts at the community level but has not been studied as much as other effects with lower impact scores.

Predicting which species can negatively affect natural ecosystems would benefit the effective control of invaders. For nonnative birds, both taxonomic relationships and species traits appear to be related to the magnitude of environmental and economic impacts [2,10,11]. In this study we did not find differences in impact scores among families, which could reinforce the idea that impacts are mostly associated with specific traits [11]. However, this study allowed us to identify species with highest local impact: *Anas platyrhinchos* (mallard), *Pycnonotus jocosus* (red-whiskered bulbul), *Garrulax canorus* (melodious laughing thrush), *Leiothrix lutea* (red-billed leiothrix), *Zosterops japonicus* (Japanese white-eye), *Z*. *lateralis* (silver-eye) and *Turdus merula* (Eurasian blackbird); see S2 Table. These species have a strong intrinsic ability to affect ecosystems and should thus be prioritized for control or eradication wherever they are introduced. Species with the highest global impact were *A*. *platyrhinchos*, *Acridotheres tristis* (common myna), and *P*. *jocosus*. Among these species we found one of the three species included on the list of 100 of the world´s worst invasive alien species (*A*. *tristis*) and one congener of another (*P*. *jocosus*) [19]. Our results show that *A*. *platyrhinchos* may also be considered among the worst invasive nonnative birds in terms of impact. We expect that global impacts of species will be associated with the number of studies for each species, but the combination of local and global impacts may be informative about how strong and widespread impacts are for a species of interest and then used as a tool to help prioritize species for control.

Introduced birds affect invaded systems at different levels: individual, population, and community levels. The scoring-system we used describes increasing levels of impact for each mechanism of impact. Thus, a score of 0 refers to a non-discernible impact, scores of 1 and 2 are for changes at the individual level, a score of 3 is for changes at the population level and scores of 4 and 5 are for changes at the community level. Some mechanisms of impact modify ecosystem properties, such as chemical impact and interaction with other non-native species.

In our analysis hybridization, disease transmission, and interaction with other non-native species were the most highly ranked impacts, suggesting that they constitute the most important threats at the population and community levels. Hybridization, the highest-scored impact in our synthesis, was highly concentrated in the families Anatidae and Phasianidae (Fig 3). In birds hybridization is an important threat, not only because of its spatial extent in some species but also owing to the production of fertile, backcrossing offspring. This makes hybridization very difficult to stop once started, and it may become more serious through time [20]. A recent review also points out that hybridization is the most important threat from introduced to native birds [6]. We found several examples of hybridization in the literature, but an exceptional case is that of the mallard, which hybridizes with several species in five regions of the world and seriously threatens some of them, such as New Zealand´s grey duck (*Anas superciliosa*) [21].

The second most highly scored category of impact was disease transmission, and it was widespread among several bird families (Fig 3). Pathogens may threaten vulnerable species by causing widespread mortality. An example is the extinction of several endemic Hawaiian birds after the introduction of the malaria blood parasite and its mosquito vector. The propagation of the disease to native birds was exacerbated by introduced species acting as reservoirs for the parasite (van Riper III *et al*., 1986; van Riper III, 1991). Non-lethal pathogens can also be important at a population level. If deleterious effects are more severe in native species than in introduced host species, apparent competition [22,23] may cause native species to decline. This is the case for the introduced ring-necked pheasant (*Phasianus colchicus*) and native grey partridge (*Perdix perdix*) acting as hosts of *Heterakis gallinarum* in Great Britain [23,24,25].

Interaction with other non-native species was the third most highly scored category of impact and was also common among nonnative birds of several families (Fig 3), owing to the high number of frugivorous birds introduced. Surprisingly, the impact that this interaction can have on native ecosystems–changes in patterns of seed dispersal–has not been taken into account in the majority of bird impact analyses. The overall impact of a nonnative bird on seed dispersal is difficult to measure because of the complexity of the system, which usually includes several species of seed dispersers and plants. If the species modifies patterns of seed deposition, it can alter the floristic composition of the environment. Assemblages of nonnative frugivorous animals do not necessarily replace the ecological function of extinct or declining populations of native seed dispersers [26,27,28,29]. Many introduced frugivores are important dispersers of nonnative plants, for example *Z*. *japonicus* in Mauritius and *T*. *merula* in New Zealand [30,31]. This positive interaction between introduced species may facilitate the invasion and exacerbate the impact of the species involved [7,32].

Although competition was the impact studied most, its average score was relatively low. This fact suggests that, even when competition is common, it may not seriously threaten native bird populations and communities. An exception is the case of *Z*. *japonicus* invading restored forests in Hawaii, which produced an abrupt decline of a population of *Loxops coccineus* (akepa) through exploitative competition and which was also associated with the decline of several other native birds [33,34]. Blackburn *et al*. [5] reviewed available evidence on competition impact and arrived at a similar conclusion.

Predation was one of the weakest impacts of nonnative birds (average impact score 1.75). Predatory birds usually eat eggs of native bird species, but predation on adult birds is important in some species [35]. Mammals are usually more important predators than birds [36,37], but it is necessary to know the relative importance of predation by nonnative birds and population trends of prey species to understand their actual importance.

Reports of chemical impact on ecosystem and grazing/ herbivory/ browsing are restricted to members of the family Anatidae. These impacts appear to be slight, with the possible exception of eutrophication of fresh water lakes caused by the increased input of nutrients via *Branta canadensis* (Canada goose) droppings [38,39]. This and other introduced waterfowl species reaching high population densities can act as novel sources of nutrients altering the biological balance of water bodies and ecosystem composition [40].

We must be aware that new invasions may continue to emerge, as many introductions are recent and species can require a long time to become invasive [41]. Also, non-native species may continue to spread and cause potentially greater impacts, helped by current climatic and environmental change [42,43,44]. In our study, residence time was not correlated with the severity of ecological impacts; however, it would be useful to analyze this trend at a population level. On the other hand, it is possible that stabilizing processes operate over time and that the initial dominance and negative impact of an invasive species can later be reversed [45].

The fact that we did not encounter differences between Strubbe´s impact scores and ours is important, as it shows that this procedure can be repeated with consistent results. A complete assessment of species impacts should weight impact scores according to the relevance for decision makers and stakeholders [46] to inform decisions about species management.

As stated in the Methods section, we recorded both negative and positive impacts on the local community. We found that in a few cases, nonnative birds positively affected the recipient community (Table 1). Although the low number of nonnative species with described benefits can be due in part to publication bias, we could identify several mechanisms by which benefits are provided to the ecosystems. Reported benefits are associated with the functional replacement of extinct species, facilitation of native species, and the ability to alter some habitat properties. For *Chrysolophus pictus* (golden pheasant), it was suggested that, as no negative impacts have been reported (although we found no studies), naturalized populations in Britain may be important for conservation of the species at global level, because it is declining in its native habitat. In this and other ways, some introduced species might be of conservation value [47], but see [48].

Conclusions drawn here are based on the 19% of naturalized species for which we found information. It is thus possible that bias exists in representation of species, because species studied can be those suspected to have some ecological impact. However, here we show that of the total of 213 successfully introduced species, 17% have ecological impacts reported somewhere. But even more significant is the fact that recent research has revealed that nonnative birds can have impacts at the community level and on chemical and structural properties of ecosystems. The inclusion of these levels of impact on scoring systems is an important step [16]. The key role of some introduced bird species as seed-dispersers, for example, can be important in modifying ecosystem structure. Also, the eutrophication of water bodies caused by nonnative birds that modify the inputs of organic matter is becoming a widely recognized problem [49]. Both of these effects can produce important changes in ecosystem dynamics and species composition [7,50].

The use of a scale of ecological impact applicable to several groups of introduced species will allow comparisons among them. We know that the relevance of introduced bird impacts compared with that of other taxa seems to be relatively high in some cases, according to impact scores obtained by Kumschick and Nentwig [9]. However, the mechanisms of bird impacts may differ from those of other taxa, such as mammals. Among introduced birds, for instance, hybridization and transmission of diseases are the most highly-scored impacts and predation and grazing/ herbivory/ browsing have a low score, while among introduced mammals predation of native species and grazing/ herbivory/ browsing are among the major threats to native ecosystems in Europe [51].

As noted in the introduction, controversies surround proposed eradication campaigns against birds; but they are usually about animal welfare issues, not about ecological impact. Social opposition can be a major constraint on eradication campaigns; for example the eradication of the Ruddy Duck (*Oxyura jamaicensis*) from Britain has faced opposition from bird-lovers [52]. Also, eradication of the Monk Parakeet (*Myopsitta monachus*) soon after its introduction to the United States was not possible because of controversies related to social perceptions of this bird, and as a consequence populations rapidly grew and expanded the introduced range of the species [53].

The evidence for ecological impact of bird populations for which eradication has been attempted is usually weak or non-existent. There have been 38 eradication attempts (29 successful) for 11 species of birds on 33 islands around the world for conservation purposes [data from Global Islands Invasive Vertebrate Database [54]]. Among those species, only two are reported here as species with high impact (a score of 3 or more in some category of impact): ruddy duck and common myna. The most threatening species in terms of ecological impacts have not been targets of eradication attempts, except for the myna. We should remember however that we may be more likely to observe impacts when they are at the population, community and ecosystem levels, when manager´s ability to control their spread and/or impact is very limited. Impacts observed at the individual level can suggest smaller, early populations that are more likely to be successfully eradicated or managed. Our analysis, however, suggests that impact scores across categories may not vary with residence times, (see Fig 5), but our sample size is limited since the impacts of many invaders have not been properly characterized. The early detection of invasive species and rapid response [55] is an efficient way to assign economic resources and can also help avoid social opposition, as most people will not yet be aware of the species presence.

This study suggests that introduced birds can have major ecological impacts at several levels, and decisions about which species are of priority for eradication should take this fact into account. We suggest that research on impact of invasive birds should focus on impacts like transmission of diseases, hybridization, and interaction with other non-native species, especially when such impacts may have reached community and ecosystem levels. Also, many families of birds that have not been studied should be prioritized for research, as well as regions other than Europe and Australasia.

## Supporting Information

S1 Fig PRISMA flow diagram. The flow diagram depicts the flow of information through the different phases of our bibliographic search. It maps out the number of records identified, included and excluded, and the reasons for exclusions (www.prisma-statement.org).
(DOC)

S1 File Scoring system for impacts of alien birds. Scoring system used to describe the severity of ecological impacts. Adapted from [Kumschick, S. and W. Nentwig. 2010. Some alien birds have as severe an impact as the most effectual alien mammals in Europe. Biological Conservation **143**:2757–2762]; and [Blackburn TM, Essl F, Evans T, Hulme PE, Jeschke JM, et al. (2014) A unified classification of alien species based on the magnitude of their environmental impacts. PLoS biology 12: e1001850].
(DOC)

S2 File List of analyzed articles. (DOC)

S1 Table List of naturalized families and species. Complete list of naturalized species included in the search. Species for which we found impact information published are marked with an asterisk.
(DOC)

S2 Table Scores of impact. Detailed scores of impact assigned to each bird species per region and category of impact. Between parentheses is indicated the source, according to the list provided in S2 File. ND = no data
(DOC)
