# Supplementary material for: Impact of Non-Native Birds on Native Ecosystems: A Global Analysis
Source: PLoS One. 2015 Nov 17;10(11):e0143070. doi: 10.1371/journal.pone.0143070 (PMC4648570; doi:10.1371/journal.pone.0143070)

**S1 Fig. PRISMA flow diagram.** The flow diagram depicts the flow of information through the different phases of our bibliographic search. It maps out the number of records identified, included and excluded, and the reasons for exclusions (www.prisma-statement.org).


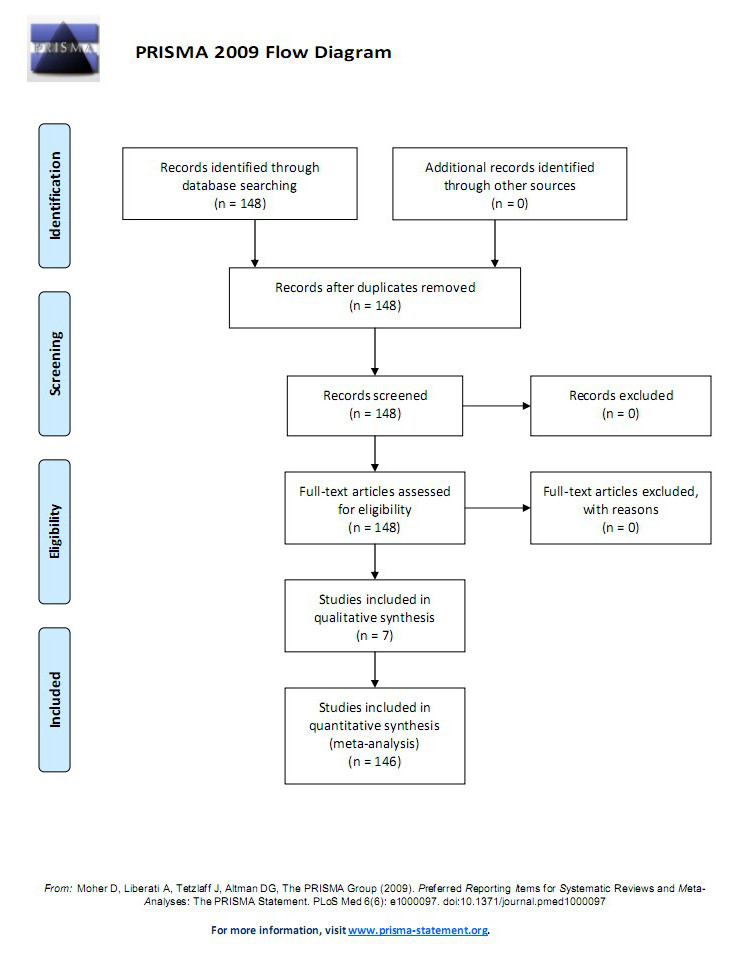

Supplement: S1 Fig — The flow diagram depicts the flow of information through the different phases of our bibliographic search. It maps out the number of records identified, included and excluded, and the reasons for exclusions (www.prisma-statement.org). (DOC) [file pone.0143070.s001.doc]
