# Supplementary material for: Impact of Non-Native Birds on Native Ecosystems: A Global Analysis
Source: PLoS One. 2015 Nov 17;10(11):e0143070. doi: 10.1371/journal.pone.0143070 (PMC4648570; doi:10.1371/journal.pone.0143070)
Supplement: S1 File — Scoring system used to describe the severity of ecological impacts. Adapted from [Kumschick, S. and W. Nentwig. 2010. Some alien birds have as severe an impact as the most effectual alien mammals in Europe. Biological Conservation 143:2757–2762]; and [Blackburn TM, Essl F, Evans T, Hulme PE, Jeschke JM, et al. (2014) A unified classification of alien species based on the magnitude of their environmental impacts. PLoS biology 12: e1001850]. (DOC) [file pone.0143070.s002.doc]

**S1 File. Scoring system for impacts of alien birds.** Scoring system used to describe the severity of ecological impacts. Adapted from [Kumschick, S. and W. Nentwig. 2010. Some alien birds have as severe an impact as the most effectual alien mammals in Europe. Biological Conservation **143**:2757-2762]; and [Blackburn TM, Essl F, Evans T, Hulme PE, Jeschke JM, et al. (2014) A unified classification of alien species based on the magnitude of their environmental impacts. PLoS biology 12: e1001850].

**1. Environmental impact**

**1.1 Grazing/Herbivory/Browsing**

0 No impact known or detectable.

1 Similar impact as native species, no major damage to plants reported.

2 Similar impact as native species, recorded negative impact on flora, impact only on abundant species.

3 Generalist herbivore, impact through unselective grazing on plants adapted to grazing, limited damages to trees, minor changes in plant communities with impact on endemic species, negative impact on seed dispersal.

4 Grazing and damage to trees by bark stripping and/or antler rubbing, damage to endemic species, recorded vegetation change reversible.

5 Grazing in areas not adapted to large herbivores, e.g. island ecosystems, high damage through bark stripping and/or antler rubbing, threat to endemic and plant species listed as vulnerable, endangered or critically endangered by IUCN, local extinctions or permanent community changes.

**1.2 Competition**

0 No impact known or detectable.

1 Very low level of competition with at least one native species, exploitation competition.

2 Competition with several native species by exploitation competition, without large impact on affected species or decline of their populations.

3 Competition with several species for food and/or space, interference competition, at least one native species declining.

4 Competition with many native species, several declining in population size, competition for food and/or space, behavioural changes in out-competed species.

5 Competes with species listed as vulnerable, endangered or critically endangered by IUCN, decline of these species, replacement or even extinction of species.

**1.3 Predation**

0 No impact known or detectable.

1 Predation known but negligible, no decline of native species.

2 Predation on several abundant species, without large impact on affected species or decline of their populations.

3 Decline of one to several native species recognized, no changes in food web structure reported.

4 Decline of many species, indirect impact by mesopredator release, changes in the food web.

5 Preys also on endemic or species listed as vulnerable, endangered or critically endangered by IUCN, local extinction.

**1.4 Transmission of diseases to wildlife**

0 No impact known or detectable.

1 Host for non-specific parasites, occasional transmission of more or less harmless diseases to one native species. No population decline in native species.

2 Occasional transmission of more or less harmless diseases, several native species affected. No or only minor population decline in native species.

3 Many native species affected, frequent transmission of more or less harmless diseases or harmful diseases transmitted to one native species. Minor population decline in native species.

4 Transmits harmful diseases to several native species or more or less harmless diseases to endemic or species listed as vulnerable, endangered or critically endangered by IUCN. Moderate population decline in native species.

5 Transmits harmful diseases to many species and/or species listed as vulnerable, endangered or critically endangered by IUCN by direct transmission, decline of these species or extinction.

**1.5 Hybridisation**

0 No impact known or detectable.

1 Hybridisation possible in captivity, but only rarely in the wild.

2 Hybridisation is more common in the wild, no offspring, but constraints to normal mating, or with offspring, but not fertile.

3 Hybridisation is more common, fertile offspring.

4 Widespread hybridisation with fertile offspring.

5 Risk of extinction of endangered species.

**1.6 Chemical, physical, or structural impact on ecosystem**

0 No impact known or detectable.

1 Slight pollution of water bodies with possible eutrophication. Slight indication of impact on successional processes.

2 Damage of vegetation resulting in bank erosion, eutrophication of water bodies infrequent.

3 Eutrophication of water bodies more frequent, change in aquatic fauna and flora, soil compaction through trampling. Impact on successional processes, infrequently, medium intensity.

4 Severe erosion processes. Eutrophication of water bodies leading to decline of species and/or change in aquatic species composition. Strong impact on successional processes.

5 Eutrophication of water bodies leading to drastic decline of species and/or change in aquatic species composition, endemic species and/or species listed as vulnerable, endangered or critically endangered by IUCN affected. Strong impact on successional processes leads to loss of habitat characteristics, damage of sites of conservation importance.

**1.7 Interaction with other non-native species**

0 No impact known or detectable.

1 Dispersal of seeds of non-native plants but with minimal effects on native species; reduction of fitness of native individuals is not detectable.

2 Dispersal of seeds of non-native plants affects fitness of native specie´s individuals without decline of their populations.

3 Dispersal of seeds of non-native plants facilitates a decline of population size of at least one native species, but no changes in community composition.

4 Dispersal of seeds of non-native plants facilitates local or population extinction of at least one native species, and produces changes in community composition that are reversible but would not have occurred in the absence of the species.

5 Dispersal of seeds of non-native plants facilitates replacement or local extinction of one or several native species, and produces irreversible changes in community composition that would not have occurred in the absence of the species.
