# Supplementary material for: Impact of Non-Native Birds on Native Ecosystems: A Global Analysis
Source: PLoS One. 2015 Nov 17;10(11):e0143070. doi: 10.1371/journal.pone.0143070 (PMC4648570; doi:10.1371/journal.pone.0143070)
Supplement: S2 File — (DOC) [file pone.0143070.s003.doc]

**S2 File. List of analyzed articles**

1. Allan, J. R., J. S. Kirby, and C. J. Feare. 1995. The biology of Canada geese *Branta canadensis* in relation to the management of feral populations. Wildlife Biology **1**:129-143.
2. Amano, H. E. and K. Eguchi. 2002. Foraging niches of introduced red-billed leiothrix and native species in Japan. ORNITHOLOGICAL SCIENCE **1**:123-131.
3. Amaral, A. J., A. B. Silva, A. R. Grosso, L. Chikhi, C. Bastos-Silveira, and D. Dias. 2007. Detection of hybridization and species identification in domesticated and wild quails using genetic markers. Folia Zoologica **56**:285.
4. Andersen, L. W. and J. Kahlert. 2012. Genetic indications of translocated and stocked grey partridges (*Perdix perdix*): does the indigenous Danish grey partridge still exist? Biological Journal of the Linnean Society **105**:694-710.
5. Balmer, D., S. Browne, and M. Rehfisch. 1996. A year in the life of golden pheasants *Chrysolophus pictus*. Pages 87-93 *in* J. S. Holmes and J. R. Simons, editors. The introduction and naturalisation of birds. The Stationery Office, London.
6. Baratti, M., M. Ammannati, C. Magnelli, and F. Dessì-Fulgheri. 2004. Introgression of chukar genes into a reintroduced red-legged partridge (*Alectoris rufa*) population in central Italy. Animal Genetics **36**:29-35.
7. Barbanera, F., G. Forcina, M. Guerrini, and F. Dini. 2011. Molecular phylogeny and diversity of the Corsican red-legged partridge: hybridization and management issues. Journal of Zoology **285**:56-65.
8. Barbanera, F., M. Guerrini, A. Khan, P. Panayides, P. Hadjigerou, C. Sokos, S. Gombobaatar, S. Samadi, B. Khan, S. Tofanelli, G. Paoli, and F. Dini. 2009a. Human-mediated introgression of exotic chukar (*Alectoris chukar*, Galliformes) genes from East Asia into native Mediterranean partridges. Biological Invasions **11**:333-348.
9. Barbanera, F., C. Marchi, M. Guerrini, P. Panayides, C. Sokos, and P. Hadjigerou. 2009b. Genetic structure of Mediterranean chukar (*Alectoris chukar*, Galliformes) populations: conservation and management implications. Naturwissenschaften **96**:1203-1212.
10. Barbanera, F., J. J. Negro, G. Di Giuseppe, F. Bertoncini, F. Cappelli, and F. Dini. 2005. Analysis of the genetic structure of red-legged partridge (*Alectoris rufa*, Galliformes) populations by means of mitochondrial DNA and RAPD markers: a study from central Italy. Biological Conservation **122**:275-287.
11. Barbanera, F., O. R. W. Pergams, M. Guerrini, G. Forcina, P. Panayides, and F. Dini. 2010. Genetic consequences of intensive management in game birds. Biological Conservation **143**:1259-1268.
12. Barilani, M., A. Bernard-Laurent, N. Mucci, C. Tabarroni, S. Kark, J. A. Perez Garrido, and E. Randi. 2007a. Hybridisation with introduced chukars (*Alectoris chukar*) threatens the gene pool integrity of native rock (*A. graeca*) and red-legged (*A. rufa*) partridge populations. Biological Conservation **137**:57-69.
13. Barilani, M., S. Deregnaucourt, S. Gallego, L. Galli, N. Mucci, R. Piombo, M. Puigcerver, S. Rimondi, J. D. Rodríguez-Teijeiro, S. Spanò, and E. Randi. 2005. Detecting hybridization in wild (*Coturnix c. coturnix*) and domesticated (*Coturnix c. japonica*) quail populations. Biological Conservation **126**:445-455.
14. Barilani, M., A. Sfougaris, A. Giannakopoulos, N. Mucci, C. Tabarroni, and E. Randi. 2007b. Detecting introgressive hybridisation in rock partridge populations (*Alectoris graeca*) in Greece through bayesian admixture analyses of multilocus genotypes. Conservation Genetics **8**:343-354.
15. Bartuszevige, A. and D. Gorchov. 2006. Avian seed dispersal of an invasive shrub. Biological Invasions **8**:1013-1022.
16. Bechard, M. and J. Bechard. 1996. Competition for nest boxes between American kestrels and European starlings in an agricultural area of southern Idaho. Pages 155-164 *in* D. Bird, D. Varland, and J. Negro, editors. Raptors in human landscapes. Academic Press, London.
17. Bennett, W. A. 1990. Scale of investigation and the detection of competition: an example from the house sparrow and house finch introductions in North America. American Naturalist **135**:725-747.
18. Blanco-Aguiar, J. A., P. González-Jara, M. E. Ferrero, I. Sánchez-Barbudo, E. Virgós, R. Villafuerte, and J. A. Dávila. 2008. Assessment of game restocking contributions to anthropogenic hybridization: the case of the Iberian red-legged partridge. Animal Conservation **11**:535-545.
19. Blanvillain, C., J. M. Salducci, G. Tutururai, and M. Maeura. 2003. Impact of introduced birds on the recovery of the Tahiti Flycatcher (*Pomarea nigra*), a critically endangered forest bird of Tahiti. Biological Conservation **109**:197-205.
20. Bonter, D. N., B. Zuckerberg, and J. L. Dickinson. 2010. Invasive birds in a novel landscape: habitat associations and effects on established species. Ecography **33**:494-502.
21. Brown, C. R. 1981. The impact of starlings on purple martin populations in unmanaged colonies. American Birds **35**:266-268.
22. Butler, C. J. 2003. Population biology of the introduced rose-ringed parakeet *Psittacula krameri* in the UK. University of Oxford, Oxford.
23. Byrd, G. V., D. I. Moriarty, and B. G. Brady. 1983. Breeding biology of wedge-tailed shearwaters at Kilauea Point, Hawaii. Condor **85**:292-296.
24. Carleton, A. R. and O. T. Owre. 1975. The Red-whiskered bubul in Florida: 1960-1971. The Auk **92**:40-57.
25. Casas, F., F. Mougeot, I. Sánchez-Barbudo, J. A. Dávila, and J. Viñuela. 2012. Fitness consequences of anthropogenic hybridization in wild red-legged partridge (*Alectoris rufa*, Phasianidae) populations. Biological Invasions **14**:295-305.
26. Castle, M. D. and B. M. Christensen. 1990. Hematozoa of wild turkeys from the Midwestern United States: translocation of wild turkeys and its potential role in the introduction of *Plasmodium kempi*. Journal of Wildlife Diseases **26**:180-185.
27. Central Coast Indian Myna Action Group. 2003. Impact of the Common (Indian) Myna on health and the environment and recommendations for the implementation of a control program. Canberra.
28. Čížková, D., V. Javürková, J. Champagnon, and J. Kreisinger. 2012. Duck´s not dead: Does restocking with captive bred individuals affect the genetic integrity of wild mallard (*Anas platyrhynchos*) population? Biological Conservation **152**:231-240.
29. Clergeau, P., D. Fourcy, S. Reeber, and P. Yésou. 2010. New but nice? Do alien sacred ibises *Threskiornis aethiopicus* stabilize nesting colonies of native spoonbills *Platalea leucorodia* at Grand-Lieu Lake, France? Oryx **44**:533-538.
30. Clergeau, P. and P. Yésou. 2006. Behavioural flexibility and numerous potential sources of introduction for the sacred ibis: causes of concern in western Europe? Biological Invasions **8**:1381-1388.
31. Cole, F. R., L. L. Loope, A. C. Medeiros, J. A. Raikes, and C. S. Wood. 1995. Conservation implications of introduced game birds in high-elevation Hawaiian shrubland. Conservation Biology **9**:306-313.
32. Czajka, C., M. P. Braun, and M. Wink. 2011. Resource use by non-native ring-necked parakeets (*Psittacula krameri*) and native starlings (*Sturnus vulgaris*) in central Europe. Open Ornithology Journal **4**:17-22.
33. Chazara, O., F. Minvielle, D. Roux, B. Bed´hom, K. Feve, J.-L. Coville, B. B. Kayang, S. Lumineau, A. Vignal, J.-M. Boutin, and X. Rognon. 2010. Evidence for introgressive hybridization of wild common quail (*Coturnix coturnix*) by domesticated Japanese quail (*Coturnix japonica*) in France. Conservation Genetics **11**:1051-1062.
34. Chimera, C. G. 2004. Investigating seed dispersal and seed predation in a Hawaiian dry forest community: implications for conservation and management. University of Hawaii, Manoa.
35. Chimera, C. G. and D. R. Drake. 2010. Patterns of seed dispersal and dispersal failure in a Hawaiian dry forest having only introduced birds. Biotropica **42**:493-502.
36. Derégnaucourt, S., J. C. Guyomarc’h, and S. Spanò. 2005. Behavioural evidence of hybridization (Japanese×European) in domestic quail released as game birds. Applied Animal Behaviour Science **94**:303-318.
37. Dhami, M. K. and B. Nagle. 2009. Review of the biology and ecology of the common myna (*Acridotheres tristis*) and some implications for management of this invasive species. Auckland.
38. Eguchi, K. and H. E. Amano. 2002. Foraging niches of introduced red-billed leiothrix and native species in Japan. ORNITHOLOGICAL SCIENCE **1**:123-131.
39. Emura, N., K. Kawakami, T. Deguchi, and K. Sone. 2012. Potential role of frugivorous birds in the recovery process of forest vegetation after feral goat eradication in Mukojima Island, the Bonin Islands. Journal of Forest Research **17**:352-359.
40. Fisher, R. J. and K. L. Wiebe. 2006. Nest site attributes and temporal patterns of northern flicker nest loss: effects of predation and competition. Oecologia **147**:744-753.
41. Fitzsimons, J. 2006. Anti-predator aggression in the common myna *Acridotheres tristis*. Australian Field Ornithology **23**:202-205.
42. Foster, J. T. and S. K. Robinson. 2007. Introduced birds and the fate of Hawaiian rainforests. Conservation Biology **21**:1248-1257.
43. Fowler, A. C., J. M. Eadie, and A. Engilis Jr. 2009. Identification of endangered Hawaiian ducks (*Anas wyvilliana*), introduced North American mallards (*A. platyrhynchos*) and their hybrids using multilocus genotypes. Conservation Genetics **10**:1747-1758.
44. Gillespie, G. D. 1985. Hybridization, introgression, and morphometric differentiation between mallard (*Anas platyrhynchos*) and grey duck (*Anas superciliosa*) in Otago, New Zealand. The Auk **102**:459-469.
45. Gleadow, R. 1982. Invasion by *Pittosporum undulatum* of the forests of Central Victoria. II. Dispersal, germination and establishment. Australian Journal of Botany **30**:185-198.
46. González-Acuña, D., A. Daugschies, K. Pohlmeyer, L. Rubilar-Contreras, O. Skewes-Ramm, E. Mey, and E. Casanueva. 2003. Ectoparásitos de la codorniz (*Callipepla californica*) en la provincia de Ñuble, Chile y su correlacion con el sexo, edad y habitat de captura. Lundiana **4**:129-134.
47. Gottdenker, N. L., T. Walsh, H. Vargas, J. Merkel, G. U. Jiménez, R. E. Miller, M. Dailey, and P. G. Parker. 2005. Assessing the risks of introduced chickens and their pathogens to native birds in the Galápagos Archipelago. Biological Conservation **126**:429-439.
48. Green, A. J. and S. Anstey. 1992. The status of the white-headed duck *Oxyura leucocephala*. Bird Conservation International **2**:185-200.
49. Guerrini, M. and F. Barbanera. 2009. Noninvasive genotyping of the red-legged partridge (*Alectoris rufa*, Phasianidae): semi-nested PCR of mitochondrial DNA from feces. Biochemical Genetics **47**:873-883.
50. Harper, M. J., M. A. McCarthy, and R. van der Ree. 2005. The use of nest boxes in urban natural vegetation remnants by vertebrate fauna. Wildlife Research **32**:509-516.
51. Heptonstall, R. E. A. 2010. The distribution and abundance of myna birds (*Acridotheres tristis*) and Rimatara lorikeets (*Vini kuhlii*) on Atiu, Cook Islands. The University of Leeds, Leeds.
52. Holzapfel, C., N. Levin, O. Hatzofe, and S. Kark. 2006. Colonisation of the Middle East by the invasive common myna *Acridotheres tristis* L., with special reference to Israel. Sandgrouse **28**:44-51.
53. Hugues, B. 1996. The ruddy duck *Oxyura jamaicensis* in the western Palearctic and the threat to the white-headed duck *Oxyura leucocephala*. Pages 79-86 *in* J. S. Holmes and J. R. Simons, editors. The introduction and naturalisation of birds. The Stationery Office, London.
54. Ingold, D. J. 1989. Nesting phenology and competition for nest sites among red-headed and red-bellied woodpeckers and European starlings. The Auk **106**: 209-217.
55. Ingold, D. J. 1994. Influence of nest-site competition between European starlings and woodpeckers. The Wilson Bulletin **106**:227-241.
56. Ingold, D. J. 1998. The influence of starlings on flicker reproduction when both naturally excavated cavities and artificial nest boxes are available. The Wilson Bulletin **110**:218-225.
57. Innes, J., E. B. Spurr, G. C. Arnold, D. Morgan, J. R. Waas, and C. Watts. 2012. Using five-minute bird counts to study magpie (*Gymnorhina tibicen*) impacts on other birds in New Zealand. New Zealand Journal of Ecology **36**:0-0.
58. Jackson, J. A. and J. Tate Jr. 1974. An analysis of nest box use by purple martins, house sparrows, and starlings in eastern North America. The Wilson Bulletin **86**:435-449.
59. Kawakami, K. and H. Higuchi. 2003a. Interspecific interactions between the native and introduced white-eyes in the Bonin Islands. Ibis **145**:583-592.
60. Kawakami, K. and H. Higuchi. 2003b. Interspecific learning by the Ogasawara Islands Honeyeater *Apalopteron familiare* from the Japanese White-eye *Zosterops japonicus* on Hahajima, the Bonin Islands, southern Japan. ORNITHOLOGICAL SCIENCE **2**:132-134.
61. Kawakami, K., L. Mizusawa, and H. Higuchi. 2009. Re-established mutualism in a seed-dispersal system consisting of native and introduced birds and plants on the Bonin Islands, Japan. Ecological Research **24**:741-748.
62. Kayser, Y., D. Clément, and M. Gauthier-Clerc. 2005. L´ ibis sacré *Threskiornis aethiopicus* sur le littoral méditerranéen français: impact sur l´ avifaune. Ornithos **12**:84-86.
63. Kerpez, T. A. and N. S. Smith. 1990. Competition between European starlings and native woodpeckers for nest cavities in saguaros. The Auk **107**:367-375.
64. Koenig, W. D. 2003. European starlings and their effect on native cavity-nesting birds. Conservation Biology **17**:1134-1140.
65. Komdeur, J. A. N. 1996. Breeding of the Seychelles magpie robin *Copsychus sechellarum* and implications for its conservation. Ibis **138**:485-498.
66. LaRosa, A. M., C. W. Smith, and D. E. Gardner. 1985. Role of alien and native birds in the dissemination of Firetree (*Myrica faya* Ait.-Myriacaceae) and associated plants in Hawaii. Pacific Science **39**:372-378.
67. Larsen, R. T., J. T. Flinders, D. L. Mitchell, and E. R. Perkins. 2007. Conservation risks of exotic chukars (*Alectoris chukar*) and their associated management: implications for a widely introduced phasianid. Wildlife Research **34**:262-270.
68. Lensink, R. 1998. Temporal and spatial expansion of the Egyptian goose *Alopochen aegyptiacus* in the Netherlands, 1967–94. Journal of Biogeography **25**:251-263.
69. Li, S.-H., C. K. L. Yeung, L. Han, M. H. Le, C.-x. Wang, P. Ding, and C.-t. Yao. 2010. Genetic introgression between an introduced babbler, the Chinese hwamei *Leucodioptron c. canorum*, and the endemic Taiwan hwamei *L. taewanus*: a multiple marker systems analysis. Journal of Avian Biology **41**:64-73.
70. Linnebjerg, J. F., D. M. Hansen, N. Bunbury, and J. M. Olesen. 2010. Diet composition of the invasive red-whiskered bulbul *Pycnonotus jocosus* in Mauritius. Journal of tropical ecology **26**:347-350.
71. Linnebjerg, J. F., D. M. Hansen, and J. M. Olesen. 2009. Gut passage effect of the introduced red-whiskered bulbul (*Pycnonotus jocosus*) on germination of invasive plant species in Mauritius. Austral Ecology **34**:272-277.
72. Lowe, K., C. Taylor, and R. Major. 2011. Do common mynas significantly compete with native birds in urban environments? Journal of Ornithology **152**:1-13.
73. Lucking, R. S. 1997. Hybridization between Madagascan red fody *Foudia madagascariensis* and Seychelles fody *Foudia sechellarum* on Aride Island, Seychelles. Bird Conservation International **7**:1-6.
74. Mandon-Dalger, I., P. Clergeau, J. Tassin, J. N. Riviere, and S. Gatti. 2004. Relationships between alien plants and an alien bird species on Reunion Island. Journal of tropical ecology **20**:635-642.
75. Martínez-Fresno, M., N. Henriques-Gil, and P. Arana. 2008. Mitochondrial DNA sequence variability in red-legged partridge, *Alectoris rufa*, Spanish populations and the origins of genetic contamination from *A. chukar*. Conservation Genetics **9**:1223-1231.
76. Meyer, J. Y. 1994. Dispersion de *Miconia calvescens* par les oiseaux dans les iles de la Societé. Pages 27-42 *in* Société d'Ornithologie de Ploynésie, editor. Seminaire Manu: Connaissance et protection des Oiseaux, Punaauia, Tahiti.
77. Millán, J., C. Gortazar, M. P. Martín-Mateo, and R. Villafuerte. 2004a. Comparative survey of the ectoparasite fauna of wild and farm-reared red-legged partridges (*Alectoris rufa*), with an ecological study in wild populations. Parasitology Research **93**:79-85.
78. Millán, J., C. Gortazar, and R. Villafuerte. 2004b. A comparison of the helminth faunas of wild and farm-reared red-legged partridge. Journal of Wildlife Management **68**:701-707.
79. Mitchell, S. F. and R. T. Wass. 1996. Grazing by black swans (*Cygnus atratus* Latham), physical factors, and the growth and loss of aquatic vegetation in a shallow lake. Aquatic Botany **55**:205-215.
80. Morgan, D., J. R. Waas, and J. Innes. 2005. Magpie interactions with other birds in New Zealand: results from a literature review and public survey. Notornis **52**:61-74.
81. Morgan, D., J. R. Waas, and J. Innes. 2006a. The relative importance of Australian magpies (*Gymnorhina tibicen*) as nest predators of rural birds in New Zealand. New Zealand Journal of Zoology **33**:17-29.
82. Morgan, D. A. I., J. R. Waas, and J. Innes. 2006b. Do territorial and non-breeding Australian magpies *Gymnorhina tibicen* influence the local movements of rural birds in New Zealand? Ibis **148**:330-342.
83. Morgan, D. K. J., J. R. Waas, J. Innes, and G. Arnold. 2012. Native bird abundance after Australian magpie (*Gymnorhina tibicen*) removal from localised areas of high resource availability. New Zealand Journal of Ecology **36**:0-0.
84. Mountainspring, S. and J. M. Scott. 1985. Interspecific competition among hawaiian forest birds. Ecological Monographs **55**:219-239.
85. Muller, W. 2008. Hybridisation, and the conservation of the grey duck in New Zealand. University of Canterbury, Canterbury.
86. Muñoz-Fuentes, V., C. Vilà, A. J. Green, J. J. Negro, and M. D. Sorenson. 2007. Hybridization between white-headed ducks and introduced ruddy ducks in Spain. Molecular Ecology **16**:629-638.
87. Newson, S. E., A. Johnston, D. Parrott, and D. I. Leech. 2011. Evaluating the population-level impact of an invasive species, ring-necked parakeet *Psittacula krameri*, on native avifauna. Ibis **153**:509-516.
88. Panayides, P., M. Guerrini, and F. Barbanera. 2011. Conservation genetics and management of the chukar partridge *Alectoris chukar* in cyprus and the Middle East. Sandgrouse **33**:34-43.
89. Parsons, H., R. E. Major, and K. French. 2006. Species interactions and habitat associations of birds inhabiting urban areas of Sydney, Australia. Austral Ecology **31**:217-227.
90. Paton, D. C., J. R. Tucker, J. B. Paton, and P. A. Paton. 1988. Avian vectors of the seeds of the European olive *Olea europaea*. South Australian Ornithologist **30**:158-159.
91. Peacock, D. S., B. J. Van Rensburg, and M. P. Robertson. 2007. The distribution and spread of the invasive alien common myna, *Acridotheres tristis* L.(Aves: Sturnidae), in southern Africa. South African Journal of Science **103**:465-473.
92. Pell, A. and C. Tidemann. 1997a. The ecology of the common myna in urban nature reserves in the Australian capital territory. Emu **97**:141-149.
93. Pell, A. S. and C. R. Tidemann. 1997b. The impact of two exotic hollow-nesting birds on two native parrots in savannah and woodland in eastern Australia. Biological Conservation **79**:145-153.
94. Phillips, R. B., H. L. Snell, and H. Vargas. 2003. Feral rock doves in the Galápagos islands: biological and economic threats. Noticias de Galápagos **62**:6-11.
95. Poling, T. D. and S. E. Hayslette. 2006. Dietary overlap and foraging competition between mourning doves and Eurasian collared-doves. Journal of Wildlife Management **70**:998-1004.
96. Puigcerver, M., D. Vinyoles, and J. D. Rodríguez-Teijeiro. 2007. Does restocking with Japanese quail or hybrids affect native populations of common quail *Coturnix coturnix*? Biological Conservation **136**:628-635.
97. Ralph, C. J. 1990. Population dynamics of land bird populations on Oahu, Hawaii: fifty years of introductions and competition. Pages 1444-1457 *in* Proc. Int. Ornithol. Congr.
98. Rebolo Ifran, N. and V. D. Fiorini. 2010. European starling (*Sturnus vulgaris*): population density and interactions with native species in Buenos Aires urban parks. ORNITOLOGIA NEOTROPICAL **21**:507-518.
99. Rehfisch, M., J. R. Allan, and G. E. Austin. 2010. The effect on the environment of Great Britain’s naturalized greater Canada *Branta canadensis* and Egyptian geese *Alopochen aegyptiacus*. BOU Proceedings – The Impacts of Non-native Species.
100. Renne, I. J., W. C. Barrow, J. Randall, A. Lori, and W. C. Bridges. 2002. Generalized avian dispersal syndrome contributes to Chinese tallow tree (*Sapium sebiferum*, Euphorbiaceae) invasiveness. Diversity and Distributions **8**:285-295.
101. Renne, I. J., S. A. Gauthreaux Jr, and C. A. Gresham. 2000. Seed dispersal of the Chinese tallow tree (*Sapium sebiferum* (L.) Roxb.) by birds in coastal South Carolina. The American Midland Naturalist **144**:202-215.
102. Rhymer, J. M., M. J. Williams, and M. J. Braun. 1994. Mitochondrial analysis of gene flow between New Zealand mallards (*Anas platyrhynchos*) and grey ducks (*A. superciliosa*). The Auk **111**:970-978.
103. Rizzo, F. 2010. Utilización de nidos de hornero (*Furnarius rufus*) por el estornino pinto (*Sturnus vulgaris*). Nuestras Aves **55**:33-35.
104. Rodríguez-Teijeiro, J. D. and M. Puigcerver. 2006. Estudio del grado de hibridación entre la codorniz común (*Coturnix coturnix*) y la codorniz japonesa (*Coturnix japonica*) en Cataluña. Generalitat de Catalunya.
105. Sanchez-Donoso, I., C. Vilà, M. Puigcerver, D. Butkauskas, J. R. C. de la Calle, P. A. Morales-Rodríguez, and J. D. Rodríguez-Teijeiro. 2012. Are farm-reared quails for game restocking really common quails (*Coturnix coturnix*)?: a genetic approach. PloS one **7**:e39031.
106. Sanders, M. D. and R. F. Maloney. 2002. Causes of mortality at nests of ground-nesting birds in the upper Waitaki basin, South Island, New Zealand: a 5-year video study. Biological Conservation **106**:225-236.
107. Scandura, M., L. Iacolina, M. Apollonio, F. Dessì-Fulgheri, and M. Baratti. 2010. Current status of the Sardinian partridge (*Alectoris barbara*) assessed by molecular markers. European Journal of Wildlife Research **56**:33-42.
108. Sherman, J. A. and P. L. Fall. 2010. Observations on feeding frequencies among native and exotic birds and fruit bats at *Erythrina variegata* and *Dysoxylum* trees on American Samoa. Pages 101-113 *in* S. G. Haberle, J. Stevenson, and M. Prebble, editors. Altered Ecologies: Fire, Climate and Human Influence on Terrestrial Landscapes. ANU E Press, Canberra.
109. Spotswood, E. N., J.-Y. Meyer, and J. W. Bartolome. 2012. An invasive tree alters the structure of seed dispersal networks between birds and plants in French Polynesia. Journal of Biogeography **39**:2007-2020.
110. Staddon, S. C., S. G. Compton, and A. Portch. 2010. Dispersal of fig seeds in the Cook Islands: introduced frugivores are no substitutes for natives. Biodiversity and Conservation **19**:1905-1916.
111. Strubbe, D. and E. Matthysen. 2007. Invasive ring-necked parakeets *Psittacula krameri* in Belgium: habitat selection and impact on native birds. Ecography **30**:578-588.
112. Strubbe, D. and E. Matthysen. 2009a. Experimental evidence for nest-site competition between invasive ring-necked parakeets (*Psittacula krameri*) and native nuthatches (*Sitta europaea*). Biological Conservation **142**:1588-1594.
113. Strubbe, D. and E. Matthysen. 2009b. Predicting the potential distribution of invasive ring-necked parakeets *Psittacula krameri* in northern Belgium using an ecological niche modelling approach. Biological Invasions **11**:497-513.
114. Strubbe, D. and E. Matthysen. 2010. The invasion of ring-necked parakeet (*Psittacula krameri*) in Europe and Belgium: mechanisms and consequences for native biota. . Pages 53-58 *in* Science Facing Aliens. Proceedings of a scientific meeting on Invasive Alien Species, Brussels, May 11^th^ 2009. Belgian Biodiversity Platform.
115. Strubbe, D., E. Matthysen, and C. H. Graham. 2010. Assessing the potential impact of invasive ring-necked parakeets *Psittacula krameri* on native nuthatches *Sitta europeae* in Belgium. Journal of Applied Ecology **47**:549-557.
116. Tassin, J., J.-N. Rivière, and P. Clergeau. 2007. Reproductive versus vegetative recruitment of the invasive tree *Schinus terebenthifolius*: implications for restoration on Reunion Island. Restoration Ecology **15**:412-419.
117. Tejedor, M. T., L. V. Monteagudo, S. Mautner, E. Hadjisterkotis, and M. V. Arruga. 2007. Introgression of *Alectoris chukar* genes into a Spanish wild *Alectoris rufa* population. Journal of Heredity **98**:179-182.
118. Tompkins, D. M., R. A. H. Draycott, and P. J. Hudson. 2000a. Field evidence for apparent competition mediated via the shared parasites of two gamebird species. Ecology Letters **3**:10-14.
119. Tompkins, D. M., J. V. Geenman, and P. J. Hudson. 2001. Differential impact of a shared nematode parasite on two gamebird hosts: implications for apparent competition. Parasitology **122**:187-193.
120. Tompkins, D. M., J. V. Greenman, P. A. Robertson, and P. J. Hudson. 2000b. The role of shared parasites in the exclusion of wildlife hosts: *Heterakis gallinarum* in the ring-necked pheasant and the grey partridge. Journal of Animal Ecology **69**:829-840.
121. Tompkins, D. M., R. A. Mitchell, and D. M. Bryant. 2006. Hybridization increases measures of innate and cell-mediated immunity in an endangered bird species. Journal of Animal Ecology **75**:559-564.
122. Tracey, J., B. Lukins, and C. Haselden. 2008. Lord Howe Island ducks: abundance, impacts and management options. Invasive Animals Cooperative Research Centre, Canberra.
123. Troetschler, R. G. 1976. Acorn woodpecker breeding strategy as affected by starling nest-hole competition. The Condor **78**:151-165.
124. Uyehara, K. J., A. Engilis, and M. H. Reynolds. 2007. Hawaiian duck's future threatened by feral mallards. US Geological Survey, Pacific Island Ecosystems Research Center.
125. Villanúa, D., F. Casas, J. Viñuela, C. Gortázar, and M. Morales. 2007. First occurrence of *Eucoleus contortus* in a little bustard *Tetrax tetrax*: negative effect of red-legged partridge *Alectoris rufa* releases on steppe bird conservation? Ibis **149**:405-406.
126. Watola, G., J. R. Allan, and C. J. Feare. 1996. Problems and management of naturalised introduced Canada geese *Branta canadensis* in Britain.*in* J. S. Holmes and J. R. Simons, editors. The introduction and naturalisation of birds. HMSO, London.
127. Weitzel, N. H. 1988. Nest-site competition between the European starling and native breeding birds in northwestern Nevada. The Condor **90**:515-517.
128. Wiebe, K. L. 2003. Delayed timing as a strategy to avoid nest-site competition: testing a model using data from starlings and flickers. Oikos **100**:291-298.
129. Wilcox, R. C. 2009. Tropical island invaders: swamp harrier (*Circus approximans*) behavior and seabird predation on Mo´orea, French Polynesia. University of California - Berkeley.
130. Williams, C. L., R. C. Brust, T. T. Fendley, G. R. Tiller Jr, and O. E. Rhodes Jr. 2005. A comparison of hybridization between mottled ducks (*Anas fulvigula*) and mallards (*A. platyrhynchos*) in Florida and South Carolina using microsatellite DNA analysis. Conservation Genetics **6**:445-453.
131. Williams, P. A. 2006. The role of blackbirds (*Turdus merula*) in weed invasion in New Zealand. New Zealand Journal of Ecology **30**:285-291.
132. Williams, P. A. and B. J. Karl. 1996. Fleshy fruits of indigenous and adventive plants in the diet of birds in forest remnants, Nelson, New Zealand. New Zealand Journal of Ecology **20**:127-145.
133. Woo, E. 2008. The role of plant-bird interactions in the invasion of *Juniperus bermudiana* in Hawaii: integrating experiments, behavior, and models. Stony Brook University, New York.
134. Wootton, J. T. 1987. Interspecific competition between introduced house finch
135. Yésou, P. and P. Clergeau. 2005. Sacred ibis: a new invasive species in Europe. Birding World **18**:517-526.
136. Freed, L. A. & Cann, R. L. (2009) Negative effects of an introduced bird species on growth and survival in a native bird community. *Current Biology*, **19**, 1736-1740.
137. Freed, L. A. & Cann, R. L. (2012) Increase of an introduced bird competitor in old-growth forest associated with restoration. *NeoBiota*, **13**, 43-60.
138. Freed, L. A., Cann, R. L. & Bodner, G. R. (2008) Incipient extinction of a major population of the Hawaii akepa owing to introduced species. *Evolutionary Ecology Research*, **10**, 931-965.
139. Gorman, M. L. (1972) The origin of the avifauna of urban & suburban Suva, Fiji. *Fiji Agricultural Journal*, **34**, 35-38.
140. Jones, C. (1996) Bird introductions to Mauritius: status and relationships with native birds. *The introduction and naturalization of birds* (ed. by J.S. Holmes & J.R. Simons). The Stationery Office, London.
141. Marion, L. (2013) Is the Sacred ibis a real threat to biodiversity? Long-term study of its diet in non-native areas compared to native areas. *Comptes rendus biologies*, **336**, 207-220.
142. Orchan, Y., Chiron, F., Shwartz, A. & Kark, S. (2013) The complex interaction network among multiple invasive bird species in a cavity-nesting community. *Biological Invasions*, **15**, 429-445.
143. Spotswood, E. N., Meyer, J.-Y. & Bartolome, J. W. (2013) Preference for an invasive fruit trumps fruit abundance in selection by an introduced bird in the Society Islands, French Polynesia. *Biological Invasions*, **15**, 2147-2156.
144. Tompkins, D. M. & Jakob-Hoff, R. (2011) Native bird declines: Don´t ignore disease. *Biological Conservation*, **144**, 668-669.
145. Van Riper Iii, C., Van Riper, S. G., Goff, M. L. & Laird, M. (1986) The epizootiology and ecological significance of malaria in Hawaiian land birds. *Ecological Monographs*, **56**, 327-344.
146. Weidema, I. R. (2000) *Introduced species in the Nordic countries*, edn. Nordic Council of Ministers.
147. Wu, J. X., Delparte, D. M. & Hart, P. J. (In Press) Movement patterns of a native and non-native frugivore in Hawaii and implications for seed dispersal. *Biotropica*.
148. Young, H. G. & Rhymer, J. M. (1998) Meller's duck: A threatened species receives recognition at last. *Biodiversity and Conservation*, **7**, 1313-1323.
