# Supplementary material for: Impact of Non-Native Birds on Native Ecosystems: A Global Analysis
Source: PLoS One. 2015 Nov 17;10(11):e0143070. doi: 10.1371/journal.pone.0143070 (PMC4648570; doi:10.1371/journal.pone.0143070)
Supplement: S1 Table — Complete list of naturalized species included in the search. Species for which we found impact information published are marked with an asterisk. (DOC) [file pone.0143070.s004.doc]

**S1 Table. List of naturalized families and species. Complete list of naturalized species included in the searching. Species for which we found impact information published are marked with an asterisk.**

| **Family** | **Genus** | **Species** | **Common name** | **Status (* = studied)** |
| --- | --- | --- | --- | --- |
| Tinamidae | *Nothoprocta* | *perdicaria* | Chilean Tinamou |  |
| Struthionidae | *Struthio* | *camelus* | Ostrich |  |
| Cracidae | *Ortalis* | *vetula* | Plain Chachalaca |  |
| Numididae | *Numida* | *meleagris* | Helmeted Guineafowl |  |
| Odontophoridae | *Oreortyx* | *pictus* | Mountain Quail |  |
|  | *Callipepla* | *californica* | California Quail | ***** |
|  | *Callipepla* | *gambelii* | Gambel's Quail |  |
|  | *Colinus* | *virginianus* | Northern Bobwhite |  |
| Phasianidae | *Meleagris* | *gallopavo* | Wild Turkey | ***** |
|  | *Tetraogallus* | *himalayensis* | Himalayan Snowcock |  |
|  | *Alectoris* | *chukar* | Chukar Partridge | ***** |
|  | *Alectoris* | *barbara* | Barbary Partridge |  |
|  | *Alectoris* | *rufa* | Red-legged Partridge | ***** |
|  | *Francolinus* | *francolinus* | Black Francolin |  |
|  | *Francolinus* | *pondicerianus* | Grey Francolin |  |
|  | *Francolinus* | *erckelii* | Erckel's Francolin |  |
|  | *Perdix* | *perdix* | Grey Partridge | ***** |
|  | *Coturnix* | *coturnix* | Common Quail |  |
|  | *Coturnix* | *chinensis* | King Quail |  |
|  | *Perdicula* | *asiatica* | Jungle Bush Quail |  |
|  | *Coturnix* | *japonica* | Japanese Quail | ***** |
|  | *Coturnix* | *ypsilophora* | Brown Quail |  |
|  | *Bambusicola* | *thoracicus* | Chinese Bamboo Partridge |  |
|  | *Gallus* | *gallus* | Red Jungle Fowl | ***** |
|  | *Gallus* | *varius* | Green Junglefowl |  |
|  | *Lophura* | *leucomelanos* | Kalij Pheasant |  |
|  | *Lophura* | *nycthemera* | Silver Pheasant |  |
|  | *Syrmaticus* | *reevesii* | Reeve's Pheasant |  |
|  | *Phasianus* | *colchicus* | Common Pheasant | ***** |
|  | *Phasianus* | *versicolor* | Green Pheasant |  |
|  | *Chrysolophus* | *pictus* | Golden Pheasant | ***** |
|  | *Chrysolophus* | *amherstiae* | Lady Amherst's Pheasant |  |
|  | *Pavo* | *cristatus* | Indian Peafowl |  |
| Anatidae | *Anser* | *indicus* | Bar-headed Goose |  |
|  | *Anser* | *caerulescens* | Snow Goose |  |
|  | *Anser* | *cygnoides* | Swan Goose |  |
|  | *Anser* | *anser* | Greylag Goose |  |
|  | *Branta* | *canadensis* | Canada Goose | ***** |
|  | *Anser* | *leucopsis* | Barnacle Goose |  |
|  | *Cygnus* | *atratus* | Black Swan | ***** |
|  | *Cygnus* | *olor* | Mute Swan |  |
|  | *Alopochen* | *aegyptiaca* | Egyptian Goose | ***** |
|  | *Tadorna* | *ferruginea* | Ruddy Shelduck |  |
|  | *Cairinia* | *moschata* | Muscovy Duck |  |
|  | *Aix* | *galericulata* | Mandarin Duck |  |
|  | *Anas* | *platyrhinchos* | Mallard | ***** |
|  | *Anas* | *melleri* | Meller's Duck |  |
|  | *Anas* | *clypeata* | Northern Shoveler |  |
|  | *Netta* | *rufina* | Red-crested Pochard |  |
|  | *Oxyura* | *jamaicensis* | Ruddy Duck | ***** |
| Phoenicopteridae | *Phoenicopterus* | *ruber* | Greater Flamingo |  |
|  | *Phoenicopterus* | *chilensis* | Chilean Flamingo |  |
| Threskiornithidae | *Threskiornis* | *aethiopicus* | Sacred Ibis | ***** |
| Ardeidae | *Nycticorax* | *nycticorax* | Black-crowned Night Heron |  |
|  | *Bubulcus* | *ibis* | Cattle Egret |  |
| Cathartidae | *Cathartes* | *aura* | Turkey Vulture |  |
| Falconidae | *Milvago* | *chimango* | Chimango Caracara |  |
| Accipitridae | *Circus* | *aeruginosus* | Western Marsh Harrier |  |
|  | *Circus* | *approximans* | Swamp Harrier | ***** |
| Rallidae | *Gallirallus* | *australis* | Weka |  |
|  | *Porphyrio* | *porphyrio* | Purple Swamphen |  |
| Pteroclididae | *Pterocles* | *exustus* | Chestnut-bellied Sandgrouse |  |
| Columbidae | *Columba* | *livia* | Rock Dove | ***** |
|  | *Streptopelia* | *decaocto* | Eurasian Collared Dove | ***** |
|  | *Streptopelia* | *risoria* | Barbary Dove |  |
|  | *Streptopelia* | *picturata* | Madagascar Turtle Dove |  |
|  | *Streptopelia* | *chinensis* | Spotted Dove | ***** |
|  | *Streptopelia* | *senegalensis* | Laughing Dove |  |
|  | *Streptopelia* | *bitorquata* | Island Collared Dove |  |
|  | *Geopelia* | *striata* | Zebra Dove |  |
|  | *Columbina* | *passerina* | Common Ground Dove |  |
|  | *Chalcophaps* | *indica* | Emerald Dove |  |
|  | *Leptotila* | *jamaicensis* | Caribbean Dove |  |
|  | *Zenaida* | *macroura* | Mourning Dove |  |
| Psittacidae | *Eolophus* | *roseicapilla* | Galah |  |
|  | *Cacatua* | *sanguinea* | Little Corella |  |
|  | *Cacatua* | *goffini* | Tanimbar Corella |  |
|  | *Cacatua* | *sulphurea* | Yellow-crested Cockatoo |  |
|  | *Cacatua* | *galerita* | Sulphur-crested Cockatoo |  |
|  | *Vini* | *kuhlii* | Kuhl's Lorikeet |  |
|  | *Prosopeia* | *tabuensis* | Red Shining Parrot |  |
|  | *Platycercus* | *elegans* | Crimson Rosella |  |
|  | *Platycercus* | *eximius* | Eastern Rosella |  |
|  | *Melopsittacus* | *undulatus* | Budgerigar |  |
|  | *Eclectus* | *roratus* | Eclectus Parrot |  |
|  | *Psittacula* | *krameri* | Ring-necked parakeet | ***** |
|  | *Psittacula* | *eupatria* | Alexandrine Parakeet |  |
|  | *Agapornis* | *canus* | Grey-headed Lovebird |  |
|  | *Agapornis* | *fischeri* | Fischer's Lovebird |  |
|  | *Agapornis* | *personatus* | Yellow-collared Lovebird |  |
|  | *Ara* | *ararauna* | Blue-and-Yellow Macaw |  |
|  | *Ara* | *severus* | Chestnut-fronted Macaw |  |
|  | *Aratinga* | *acuticaudata* | Blue-crowned Parakeet |  |
|  | *Aratinga* | *mitrata* | Mitred Parakeet |  |
|  | *Aratinga* | *holochlora* | Green Parakeet |  |
|  | *Aratinga* | *erythrogenys* | Red-masked Parakeet |  |
|  | *Aratinga* | *canicularis* | Orange-fronted Parakeet |  |
|  | *Aratinga* | *pertinax* | Brown-throated Parakeet |  |
|  | *Nandayus* | *nenday* | Nanday Parakeet |  |
|  | *Myopsitta* | *monachus* | Monk Parakeet |  |
|  | *Forpus* | *passerinus* | Green-rumped Parrotlet |  |
|  | *Brotogeris* | *versicolurus* | White-winged Parakeet |  |
|  | *Brotogeris* | *chiriri* | Yellow-chevroned Parakeet |  |
|  | *Amazona* | *ventralis* | Hispaniola Parrot |  |
|  | *Amazona* | *viridigenalis* | Red-Crowned Parrot |  |
|  | *Amazona* | *finschi* | Lilac-crowned Parrot |  |
|  | *Amazona* | *oratrix* | Yellow-headed Parrot |  |
|  | *Amazona* | *ochrocephala* | Yellow-crowned Parrot |  |
|  | *Amazona* | *amazonica* | Orange-winged Parrot |  |
| Cuculidae | *Crotophaga* | *ani* | Smooth-billed Ani |  |
| Tytonidae | *Tyto* | *alba* | Barn Owl |  |
| Strigidae | *Bubo* | *virginianus* | Great Horned Owl |  |
|  | *Athene* | *noctua* | Little Owl |  |
| Apodidae | *Aerodramus* | *bartschi* | Marianas Swiftlet |  |
| Alcedinidae | *Dacelo* | *novaeguineae* | Laughing Kookaburra |  |
| Tyrannidae | *Pitangus* | *sulphuratus* | Great Kiskadee |  |
| Meliphagidae | *Manorina* | *melanocephala* | Noisy Miner |  |
| Craticidae | *Gymnorhina* | *tibicen* | Australian Magpie | ***** |
| Dicruridae | *Dicrurus* | *macrocercus* | Black Drongo |  |
| Corvidae | *Cyanocorax* | *dickeyi* | Tufted Jay |  |
|  | *Corvus* | *splendens* | Howse Crow |  |
|  | *Corvus* | *frugilegus* | Rook |  |
|  | *Corvus* | *brachyrhynchos* | American Crow |  |
|  | *Corvus* | *monedula* | Eurasian Jackdaw |  |
|  | *Pica* | *pica* | Common Magpie |  |
| Alaudidae | *Alauda* | *arvensis* | Eurasian Skylark |  |
| Pycnonotidae | *Pycnonotus* | *jocosus* | Red-whiskered Bulbul | ***** |
|  | *Pycnonotus* | *cafer* | Red-vented Bulbul | ***** |
|  | *Pycnonotus* | *aurigaster* | Sooty-headed Bulbul |  |
|  | *Pycnonotus* | *goiavier* | Yellow-vented Bulbul |  |
| Sylviidae | *Cettia* | *diphone* | Japanese Bush Warbler | ***** |
| Timaliidae | *Garrulax* | *canorus* | Melodious Laughing Thrush | ***** |
|  | *Garrulax* | *pectoralis* | Greater Necklaced Laughing Thrush |  |
|  | *Garrulax* | *caerulatus* | Grey-sided Laughing Thrush |  |
|  | *Garrulax* | *perspicillatus* | Masked Laughing Thrush |  |
|  | *Leiothrix* | *lutea* | Red-billed Leiothrix | ***** |
| Zosteropidae | *Zosterops* | *japonicus* | Japanese White-eye | ***** |
|  | *Zosterops* | *lateralis* | Silver-eye | ***** |
|  | *Zosterops* | *natalis* | Christmas Island White-eye |  |
| Mimidae | *Mimus* | *polyglottos* | Northern Mockingbird | ***** |
|  | *Mimus* | *gilvus* | Tropical Mockingbird |  |
| Sturnidae | *Gracula* | *religiosa* | Hill myna |  |
|  | *Acridotheres* | *cristatellus* | Crested Myna |  |
|  | *Acridotheres* | *fuscus* | Jungle Myna | ***** |
|  | *Acridotheres* | *javanicus* | White-vented Myna |  |
|  | *Acridotheres* | *melanopterus* | Black-winged Myna |  |
|  | *Acridotheres* | *cinereus* | Pale-bellied Myna |  |
|  | *Acridotheres* | *gingianus* | Bank Myna |  |
|  | *Acridotheres* | *tristis* | Common Myna | ***** |
|  | *Sturnus* | *vulgaris* | European Starling | ***** |
|  | *Sturnus* | *contra* | Asian Pied Starling |  |
|  | *Sturnus* | *burmannicus* | Vinous-breasted Starling | ***** |
| Turdidae | *Turdus* | *merula* | Eurasian Blackbird | ***** |
|  | *Turdus* | *philomelos* | Song Thrush | ***** |
|  | *Turdus* | *poliocephalus* | Island Thrush |  |
| Muscicapidae | *Copsychus* | *malabaricus* | White-rumped Shama |  |
| Passeridae | *Passer* | *domesticus* | House Sparrow | ***** |
|  | *Passer* | *montanus* | Eurasian Tree Sparrow |  |
|  | *Passer* | *hispaniolensis* | Spanish Sparrow |  |
| Ploceidae | *Ploceus* | *cucullatus* | Village Weaver |  |
|  | *Ploceus* | *jacksoni* | Golden-backed Weaver |  |
|  | *Ploceus* | *intermedius* | Lesser Masked Weaver |  |
|  | *Ploceus* | *manyar* | Streaked Weaver |  |
|  | *Foudia* | *madagascariensis* | Red Fody |  |
|  | *Foudia* | *sechellarum* | Seychelles Fody | ***** |
|  | *Euplectes* | *franciscanus* | Orange Bishop |  |
|  | *Euplectes* | *afer* | Yellow-crowned Bishop |  |
| Estrildidae | *Uraeginthus* | *bengalus* | Red-cheeked Cordon-bleu |  |
|  | *Uraeginthus* | *angolensis* | Blue-breasted Cordon-bleu |  |
|  | *Estrilda* | *melpoda* | Orange-cheeked Waxbill |  |
|  | *Estrilda* | *caerulescens* | Red-tailed Lavender Waxbill |  |
|  | *Estrilda* | *astrild* | Common Waxbill |  |
|  | *Estrilda* | *troglodytes* | Black-rumped Waxbill |  |
|  | *Amandava* | *amandava* | Red Avadavat |  |
|  | *Neochmia* | *temporalis* | Red-browned Finch |  |
|  | *Lonchura* | *cucullata* | Bronze Mannikin |  |
|  | *Lonchura* | *malabarica* | Indian Silverbill |  |
|  | *Lonchura* | *punctulata* | Scaly-breasted Munia |  |
|  | *Lonchura* | *leucogastroides* | Javan Munia |  |
|  | *Lonchura* | *malacca* | Black-headed Munia |  |
|  | *Lonchura* | *hunsteini* | White-cowled Mannikin |  |
|  | *Lonchura* | *castaneothorax* | Chestnut-breasted Mannikin |  |
|  | *Lonchura* | *oryzivora* | Java Sparrow |  |
|  | *Lonchura* | *striata* | White-rumped Munia |  |
| Viduidae | *Vidua* | *macroura* | Pin-tailed Whydah |  |
|  | *Vidua* | *paradisaea* | Eastern paradise |  |
| Prunellidae | *Prunella* | *modularis* | Dunnock |  |
| Fringillidae | *Fringilla* | *coelebs* | Chaffinch |  |
|  | *Serinus* | *canaria* | Island Canary |  |
|  | *Serinus* | *mozambichus* | Yellow-fronted Canary |  |
|  | *Serinus* | *canicollis* | Cape Canary |  |
|  | *Serinus* | *flaviventris* | Yellow Canary |  |
|  | *Carduelis* | *chloris* | European Greenfinch |  |
|  | *Carduelis* | *carduelis* | European Goldfinch |  |
|  | *Carduelis* | *cucullata* | Red Siskin |  |
|  | *Carduelis* | *flammea* | Common Redpoll |  |
|  | *Carpodacus* | *mexicanus* | House Finch | ***** |
| Icteridae | *Icterus* | *icterus* | Troupial |  |
|  | *Icterus* | *pectoralis* | Spot-breasted Oriole |  |
|  | *Molothrus* | *bonariensis* | Shiny Cowbird |  |
|  | *Sturnella* | *neglecta* | Western Meadowlark |  |
|  | *Quiscalus* | *lugubris* | Carib Grackle |  |
| Emberizidae | *Emberiza* | *citrinella* | Yellowhammer |  |
|  | *Emberiza* | *cirlus* | Cirl Bunting |  |
|  | *Sicalis* | *luteola* | Grassland Yellow Finch |  |
|  | *Sicalis* | *flaveola* | Saffron Finch |  |
|  | *Diuca* | *diuca* | Common Diuca Finch |  |
|  | *Tiaris* | *olivaceus* | Yellow-faced Grassquit |  |
|  | *Tiaris* | *canorus* | Cuban Grassquit |  |
|  | *Paroaria* | *coronata* | Red-crested Cardinal |  |
|  | *Paroaria* | *capitata* | Yellow-billed Cardinal |  |
| Cardinalidae | *Cardinalis* | *cardinalis* | Northern Cardinal | ***** |
| Thraupidae | *Ramphocelus* | *dimidiatus* | Crimson-backed Tanager |  |
|  | *Cyanerpes* | *cyaneus* | Red-legged Honeycreeper |  |
