# Supplementary material for: Impact of Non-Native Birds on Native Ecosystems: A Global Analysis
Source: PLoS One. 2015 Nov 17;10(11):e0143070. doi: 10.1371/journal.pone.0143070 (PMC4648570; doi:10.1371/journal.pone.0143070)
Supplement: S2 Table — Detailed scores of impact assigned to each bird species per region and category of impact. Between parentheses is indicated the source, according to the list provided in S2 File. ND = no data (DOC) [file pone.0143070.s005.doc]

**S2 Table. Scores of impact.** Detailed scores of impact assigned to each bird species per region and category of impact. Between parenthesis is indicated the source, according to the list provided in S2 File. ND=no data

| Family | Species | Continent | Competition | Hybridization | Interaction with other non-native species | Disease transmission | Predation | Grazing/ herbivory/ browsing | Chemical, physical, or structural impact on the ecosystem |
| --- | --- | --- | --- | --- | --- | --- | --- | --- | --- |
| Odontophoridae | *Callipepla californica* | South America | ND | ND | ND | 0 (47) | ND | ND | ND |
| Phasianidae | *Alectoris chukar* | Europe | ND | 4 (6-12,14, 18, 26, 49, 75, 117) | ND | ND | ND | ND | ND |
|  |  | North America | ND | ND | 0 (68) | ND | ND | ND | ND |
|  |  | Pacific Ocean | ND | ND | 0 (32) | ND | ND | ND | ND |
|  | *Alectoris rufa* | Europe | ND | 3 (88) | ND | 1 (77, 78, 125) | ND | ND | ND |
|  | *Chrysolophus pictus* | Europe | 0 (5) | ND | ND | ND | ND | ND | ND |
|  | *Coturnix japonica* | Europe | ND | 3 (3, 13, 33, 36, 96, 104, 105) | ND | ND | ND | ND | ND |
|  | *Gallus gallus* | Pacific Ocean | ND | ND | 1 (110) | 2 (47) | ND | ND | ND |
|  | *Meleagris gallopavo* | North America | ND | ND | ND | 1 (26) | ND | ND | ND |
|  | *Perdix perdix* | Europe | ND | 3 (4) | ND | ND | ND | ND | ND |
|  | *Phasianus colchicus* | Europe | ND | ND | ND | 2 (118-120) | ND | ND | ND |
|  |  | Pacific Ocean | ND | ND | 2 (31) | ND | ND | ND | ND |
| Anatidae | *Alopochen aegyptiaca* | Europe | 1 (69,100) | ND | ND | ND | ND | ND | 1 (69, 100) |
|  | *Anas platyrhinchos* | Europe | ND | 3 (29) | ND | ND | ND | ND | ND |
|  |  | Pacific Ocean | ND | 3 (44, 124, 126) | ND | ND | ND | ND | ND |
|  |  | Australasia | ND | 4,5 (45, 86, 103) | ND | ND | ND | ND | ND |
|  |  | North America | ND | 4 (133) | ND | ND | ND | ND | ND |
|  |  | Africa | ND | 1 (159) | ND | ND | ND | ND | ND |
|  | *Branta canadensis* | Europe | 1.5 (1, 100, 129, 156) | 2,5 (1,100, 156) | ND | ND | ND | 3 (156) | 2,5 (1, 100, 129) |
|  | *Cygnus atratus* | Australasia | ND | ND | ND | ND | ND | 1 (80) | ND |
|  | *Oxyura jamaicensis* | Europe | 2 (54) | 3,5 (49, 54, 87) | ND | ND | ND | ND | ND |
| Threskiornithidae | *Threskiornis aethiopicus* | Europe | 2,5 (29, 62, 135) | ND | ND | ND | 2,5 (30, 62, 135, 140) | ND | ND |
| Accipitridae | *Circus approximans* | Pacific Ocean | ND | ND | ND | ND | 1 (129) | ND | ND |
| Columbidae | *Columba livia* | Pacific Ocean | ND | ND | ND | 2,5 (94) | ND | ND | ND |
|  | *Streptopelia chinensis* | Australasia | ND | ND | 0 (90) | ND | ND | ND | ND |
|  | *Streptopelia decaocto* | North America | 0,5 (20, 95) | ND | ND | ND | ND | ND | ND |
| Psittacidae | *Psittacula krameri* | Europe | 2 (22, 32, 87, 111-115) | ND | ND | ND | ND | ND | ND |
|  |  | Asia | 1 (142) | ND | ND | ND | ND | ND | ND |
|  |  | Indian Ocean | 4 (140) | ND | ND | ND | ND | ND | ND |
| Craticidae | *Gymnorhina tibicen* | Australasia | 2,5 (57, 80, 82, 83) | ND | ND | ND | 2 (81, 106) | ND | ND |
| Pycnonotidae | *Pycnonotus cafer* | Pacific Ocean | 2 (19, 108, 139) | ND | 4 (76, 109, 143) | ND | ND | ND | ND |
|  | *Pycnonotus jocosus* | North America | ND | ND | 5 (24) | ND | ND | ND | ND |
|  |  | Indian Ocean | 4 (140) | ND | 5 (70, 74, 116) | ND | ND | ND | ND |
| Sylviidae | *Cettia diphone* | Pacific Ocean | ND | ND | 0 (42) | ND | ND | ND | ND |
| Timaliidae | *Garrulax canorus* | Pacific Ocean | 2 (84) | ND | 2 (42) | ND | ND | ND | ND |
|  |  | Asia | ND | 4,5 (69) | ND | ND | ND | ND | ND |
|  | *Leiothrix lutea* | Asia | 1 (2, 38) | ND | ND | ND | ND | ND | ND |
|  |  | Pacific Ocean | 2 (84) | ND | 2 (42) | 5 (145) | ND | ND | ND |
| Zosteropidae | *Zosterops japonicus* | Pacific Ocean | 4 (59, 60, 84, 97, 136-138) | ND | 3,5 (34, 35, 39, 42, 61, 66, 133, 147) | 5 (145) | ND | ND | ND |
|  | *Zosterops lateralis* | Pacific Ocean | ND | ND | 4,5 (76, 109) | ND | ND | ND | ND |
| Mimidae | *Mimus polyglottos* | Pacific Ocean | ND | ND | 3 (34,35) | ND | ND | ND | ND |
| Sturnidae | *Acridotheres fuscus* | Pacific Ocean | 2 (139) | ND | ND | ND | ND | ND | ND |
|  | *Acridotheres tristis* | Africa | 1,5 (91) | ND | ND | ND | ND | ND | ND |
|  |  | Australasia | 2,5 (27, 37, 41, 50, 72, 89, 92, 93) | ND | ND | ND | 2 (27, 37) | ND | ND |
|  |  | Indian Ocean | 4 (65, 140) | ND | ND | ND | ND | ND | ND |
|  |  | Asia | 1 (52, 142) | ND | ND | ND | 1 (52) | ND | ND |
|  |  | Pacific Ocean | 3 (19, 51,139) | ND | 1 (110) | ND | 2 (23) | ND | ND |
|  | *Sturnus vulgaris* | Australasia | 2 (93) | ND | 2.5 (90, 132) | ND | ND | ND | ND |
|  |  | North America | 2.5 (16, 21, 40, 54-56, 63, 64, 123, 127, 128) | ND | 2.5 (15, 100, 101) | ND | ND | ND | ND |
|  |  | South America | 1 (98, 103) | ND | ND | ND | ND | ND | ND |
|  | *Sturnus burmannicus* | Asia | 2 (142) | ND | ND | ND | ND | ND | ND |
| Turdidae | *Turdus merula* | Australasia | ND | ND | 5 (45, 90, 131, 132) | 3.5 (144) | ND | ND | ND |
|  | *Turdus philomelos* | Australasia | ND | ND | 2 (132) | ND | ND | ND | ND |
| Passeridae | *Passer domesticus* | Australasia | ND | ND | 0 (90) | ND | ND | ND | ND |
|  |  | North America | 1 (17, 58) | ND | ND | ND | ND | ND | ND |
| Ploceidae | *Foudia sechellarum* | Indian Ocean | ND | 3 (73) | ND | ND | ND | ND | ND |
| Fringillidae | *Carpodacus mexicanus* | North America | 2 (134) | ND | ND | ND | ND | ND | ND |
|  |  | Pacific Ocean | ND | ND | 2 (66, 133) | ND | ND | ND | ND |
| Cardinalidae | *Cardinalis cardinalis* | Pacific Ocean | 1 (84) | ND | 2 (34, 35, 66, 133) | ND | ND | ND | ND |
